# Loss of 5-HT_2C_ receptor function alters motor behavior in male and female mice with and without spinal cord injury

**DOI:** 10.3389/fncir.2025.1681120

**Published:** 2025-09-29

**Authors:** Margaret I. Sim, Derin Birch, Amr A. Mahrous, C.J. Heckman, Vicki M. Tysseling

**Affiliations:** ^1^Department of Physical Therapy and Human Movement Sciences, Feinberg School of Medicine Northwestern University, Chicago, IL, United States; ^2^Department of Neuroscience, Feinberg School of Medicine Northwestern University, Chicago, IL, United States; ^3^Department of Physical Medicine and Rehabilitation, Feinberg School of Medicine Northwestern University, Chicago, IL, United States

**Keywords:** serotonin, spinal cord injury, locomotion, spinal motoneurons, persistent inward currents, hyperreflexia

## Abstract

The 5-HT_2C_ receptor is involved in the regulation of spinal motor function, specifically in both volitional and involuntary motor behavior. It contributes to various aspects of voluntary movement, such as locomotion, gait, coordination, and muscle contractions. It also contributes to involuntary motor behavior (i.e., spasms), which affects many individuals with spinal cord injury. Despite its known involvement in motor function, additional research in uninjured mice is required to assess whether specific gait parameters and muscle contractility are directly linked to the 5-HT_2C_ receptor. In injured mice, further research is needed to determine whether the expression of the 5-HT_2C_ receptor is altered in the lumbar and sacral spinal cord after injury. It is also necessary to determine whether voluntary locomotion, involuntary motor behavior, or the expression of this receptor is influenced by sex, as it is unknown if there is a difference in 5-HT_2C_ receptor expression between male and female mice. The aim of this study is to investigate volitional and involuntary motor behavior of male and female uninjured and spinal cord-injured knock-out mice. Mice that express a non-functional form of the 5-HT_2C_ receptor were compared to typical-functioning wildtype mice. Volitional behavioral assessments revealed mild strength and stability deficits in the knock-out mice when compared to wildtype mice. We also compared the capacity of spinal cord tissue to generate sensory evoked activity, and it was revealed that male knock-out mice exhibited less involuntary motor behavior both *ex vivo* and *in vivo* than male wildtype mice. Western blot analysis revealed that injury status, sex, and genotype affected the relative expression of the 5-HT_2C_ receptor in both the lumbar and sacral spinal cord, with female KO mice exhibiting a compensatory mechanism post-SCI via upregulation of the 5-HT_2A_ receptor. Through a comprehensive approach combining behavioral assessments, electrophysiological experiments, and whole-tissue protein analysis, our findings provide strong evidence that the 5-HT_2C_ receptor is differentially regulated by sex, genotype, and spinal cord injury. These findings underscore the importance of considering sex as a biological variable and suggest that future therapeutic strategies targeting the 5-HT_2C_ receptor account for sex-specific differences in 5-HT_2C_ receptor expression and function.

## 1 Introduction

Serotonin (5-HT) is a complex neuromodulator that has been implicated in a variety of physiological functions (Mosher et al., 2005). In motor control, descending 5-HT within the spinal cord is responsible for the regulation of spinal motoneuron (MN) excitability (Bayliss et al., 1995; Heckman et al., 2003; Lee and Heckman, 1998; Lindsay and Feldman, 1993; Zhang et al., 1997) through the activation of 5-HT_2_ receptors. The 5-HT_2_ class of receptors are G-protein coupled receptors, of which there are three subtypes: the 5-HT_2A_, 5-HT_2B_, and 5-HT_2C_ receptors (Barnes and Sharp, 1999). All three of the 5-HT_2_ receptor subtypes have been shown to possess similar molecular structure, pharmacology, and signal transduction pathways to one another (Parajulee and Kim, 2023). Of the three 5-HT_2_ receptor subtypes, 5-HT_2A_ and 5-HT_2C_ have been the most extensively studied for their role in motor function.

Voltage-gated CaV1.3 L-type calcium channels are expressed on MNs and mediate persistent inward currents (PICs) (Binder et al., 2011). These channels can amplify synaptic inputs and generate sustained depolarization in response to brief excitatory inputs (Bennett et al., 2004; Heckman et al., 2005). The presence of serotonin and the activation of the 5-HT_2_ receptors have been shown to be essential for facilitating PICs in the intact as well as in the injured spinal cord (Hounsgaard et al., 1988; Murray et al., 2010, 2011b). Despite these potent effects that 5-HT has on MN excitability, the roles of different 5-HT_2_ receptors in motor behavior are still not fully understood.

Previous studies have shown that pharmacologically blocking the 5-HT_2C_ receptor (5-HT_2C_R) does not affect locomotion or reflexes (Majczyński et al., 2020). However, several off-target effects of the pharmacological blockers might have affected this outcome. Research using 5-HT_2C_R knock-out (KO) mice has shown conflicting results regarding the effect on locomotion in intact animals (Heisler et al., 2007; Hill et al., 2011; Nebuka et al., 2020), highlighting a need for further investigation. Importantly, the 5-HT_2C_R has been shown to be involved in the development of muscle spasms following spinal cord injury (SCI) (Murray et al., 2010; Tysseling et al., 2017), suggesting the potential for therapeutic interventions via manipulation or targeting of these receptors. It is also unknown whether these receptors mediate sex differences in normal motor behaviors or motor recovery after SCI, given the reported relationship between sex hormones (i.e., estradiol and testosterone) and serotonin receptor expression (Kugaya et al., 2003; Popova and Amstislavskaya, 2002). Henceforth, the goal of the current study is to investigate the role of the 5-HT_2C_R in motor behavior in uninjured and spinal cord-injured wildtype (WT) and genetically-modified KO mice that lack the functional 5-HT_2C_R.

Recent evidence has shown sex-specific differences in PIC generation between male and female human subjects (Jenz et al., 2023). This study found that although motor unit discharge rates did not differ between male and female healthy subjects, biological sex was a critical variable in estimating PIC magnitude. Female subjects were revealed to have larger PIC estimates in lower limb MNs than their male counterparts. In light of these findings, in combination with previous research that has shown a connection between 5-HT and sex hormones, we deemed it necessary to include sex-specific analyses across all experimental procedures conducted.

The second purpose of this study was to investigate potential differences in involuntary motor behavior post-SCI between the KO mice and their WT counterparts. This study is unique because a thoracic model of injury is used, whereas previous research has typically used a sacral transection, and involuntary motor behavior has been examined primarily in the sacrocaudal spinal cord. It is well understood that injury severity and recovery outcome are predominantly influenced by the level of SCI (Alizadeh et al., 2019; Coleman, 2004). Using a thoracic model of spinal cord transection is more clinically relevant, as approximately 35% of all SCIs occur at the thoracic level (Alizadeh et al., 2019), and it allows us to examine mechanistic alterations in the lumbar spinal cord, as the descending pathways that project to the lumbar section are responsible for hindlimb locomotion in mice (Han et al., 2019).

To further investigate mechanistic alterations underlying involuntary motor behavior post-SCI, the relative protein expression of the 5-HT_2C_R (in only WT mice) and the 5-HT_2A_R (in both WT and KO mice) in the lumbar and sacral spinal cord tissue of uninjured and injured mice was assessed using western blot. Previous research regarding the upregulation of the 5-HT_2C_R has shown conflicting results. Total mRNA studies conducted in the rat have suggested that the 5-HT_2C_R does not change post-sacral transection, but the constitutive activity of the receptor increases (Murray et al., 2010). However, western blot analyses have suggested that the 5-HT_2C_R does become upregulated in the sacral cord post-sacral transection (Ren et al., 2013). As for the 5-HT_2A_R, previous research has suggested that this receptor is also upregulated post-sacral transection (Kong et al., 2010). In line with the second objective of this study, we aim to clarify discrepancies in 5-HT_2C_R and 5-HT_2A_R protein expression within both the lumbar and sacral spinal cord to better understand the potential underlying molecular mechanisms related to involuntary motor behavior post-SCI.

## 2 Materials and methods

### 2.1 Animals

All procedures in this study were reviewed and approved by the Northwestern University Institutional Animal Care and Use Committee (IACUC) and were compliant with the National Institutes of Health Guide for the Care and Use of Laboratory Animals. Two groups of adult mice, inclusive of both sexes, were used in this study. Transgenic knockout mice, B6.129-Htr2c^tm1Jul^/J (Jackson Laboratory, Bar Harbor, ME, USA, RRID: IMSR_JAX:002627) (5-HT_2C_R KO), which lack the functional serotonin 2C receptor (Tecott et al., 1995), were compared to wild-type C57Bl/6J mice (Jackson Laboratory, Bar Harbor, ME, USA, RRID: IMSR_JAX:000664) (WT). Jackson Laboratory confirms that the B6.129-Htr2c^tm1Jul^/J mice had been backcrossed to C57BL/6J at least five times. Following behavioral testing in intact animals, both experimental groups received SCI at 10 weeks of age and were compared to uninjured littermate controls. Behavioral testing was performed before and after SCI and was followed by terminal procedures in which either proteins were extracted for western blot analysis or the sacrocaudal spinal cord was removed for *ex vivo* analysis (see below). There were a total of 107 mice used in this study, and the total n of mice in each group (i.e., male WT, female WT, male KO, and female KO), as well as the specific experiments each mouse underwent, are listed in Supplementary Tables 1–4.

### 2.2 Spinal cord injury

At ten weeks of age, mice were anesthetized with isoflurane, and a laminectomy was performed at the T10 spinal level to expose the T11-T12 spinal segments. The spinal cord was completely transected using spring scissors. Following transection, mice were evaluated for 10 weeks using the Basso Mouse Scale (see below) to measure functional motor recovery. SCI was characterized as ‘chronic’ 10 weeks post-surgery. At this point, the test subjects underwent additional behavioral testing before terminal procedures.

### 2.3 Volitional motor behavior testing

#### 2.3.1 Basso mouse scale

The Basso Mouse Scale (BMS) is an established open-field behavioral assessment used to evaluate hindlimb functional deficits following spinal cord injury in mice (Basso et al., 2006). Injured male WT (*n* = 5), female WT (*n* = 6), male KO (*n* = 8), and female KO (*n* = 7) mice underwent weekly evaluations beginning 1 day post-SCI (week 0) and continuing until the injury was chronic (week 10). The scoring of this assessment ranges from 0, indicating complete paralysis and total loss of hindlimb function, to 9, indicating standard functionality and locomotive ability. Sub-scales were not necessary to include due to low function in our mice. An average of the left and right hindlimb scores was taken to obtain the BMS score for each subject. All test subjects retained a BMS score of < 2 throughout the experiment.

#### 2.3.2 DigiGait™

Treadmill gait analysis was conducted using the DigiGait™ system (Mouse Specifics Inc., Framingham, MA) to assess specific differences in gait between uninjured adult male WT (*n* = 5), female WT (*n* = 5), male KO (*n* = 12), and female KO (*n* = 11) mice. Only five WT mice of either sex were used due to the lack of variability within control mice; however, it should be considered that the WT data may be underpowered because of this stark difference in n. The protocol for this assessment has been discussed previously (Mancuso et al., 2011). Briefly, the DigiGait™ system consisted of a transparent treadmill, horizontally fixed at 0 ° (5 cm in width, 25 cm in length), which contained the mice as they ran at a constant velocity. The velocity used in this assessment was fixed at 24 cm/s, a speed that mice maintained without veering off to one side. A high-temporal-resolution video recording (~5 s) was captured for each mouse before analysis was performed using the DigiGait™ Imaging and Analysis software v12.2 (Mouse Specifics Inc., Framingham, MA). The software utilizes coding and artificial intelligence algorithms to detect the spatial coordinates and direction of pixels, enabling it to calculate either the duration spent in each gait phase or the distance between each paw. Data was averaged between left and right paws in both the forelimbs and hindlimbs.

#### 2.3.3 Grip strength

A grip strength meter (Ugo Basile, Gemonio, Italy) was utilized to determine the maximum and average forces displayed by the mice in the forelimbs, hindlimbs, and all four limbs combined. This experimental procedure has been described previously (Papaneophytou et al., 2018). Uninjured male WT (*n* = 18), female WT (*n* = 19), male KO (*n* = 12), and female KO (*n* = 14) mice were assessed. For forelimb and all-limb testing, each mouse was held by the base of the tail and lowered onto the plastic grid to grip with its forepaws or all four paws, respectively. The mouse was then pulled away slowly, with the torso horizontal to the table, allowing the limbs to flex until the grip was released. For hindlimb testing, the mice gripped onto the triangle bar attachment with their forelimbs and were then lowered down until only the hindpaws were able to grip onto the plastic attachment. The same parallel pulling motion was used. A digital force transducer recorded the peak pull force (g). Tension was recorded when the mouse released the grip from the attachment. The order in which the mice were tested was randomized, and each mouse underwent five trials per grip type with about 30 min between each trial.

### 2.4 Involuntary motor behavior testing

#### 2.4.1 *In vivo* flexor withdrawal testing

The flexor withdrawal reflex analysis was conducted on injured male WT (*n* = 8), female WT (*n* = 11), male KO (*n* = 8), and female KO (*n* = 9) mice to evaluate hyperreflexia and muscle spasms in the hindlimb. This experimental procedure has been described in detail previously (Tysseling et al., 2017). Briefly, electromyography (EMG) recording electrodes were placed in the tibialis anterior (TA) and lateral gastrocnemius (LG) muscles of the hindlimb under isoflurane anesthesia. Ball electrodes used to deliver electrical stimulation were then affixed to the plantar side of the hind paw to evoke the flexion withdrawal reflex. When the mouse was fully awake following cessation of gaseous anesthesia, the response threshold was determined by increasing the current amplitude by 10 μA increments until an observable movement in the hindlimb could be identified. After the threshold had been determined, 10 stimuli (5 pulses, 1 ms pulse width, 100 Hz) were applied at 5x threshold with a minimum of 2 min of rest between stimuli, until a total of 5 viable recordings were obtained. A trial was considered viable when there was no substantial EMG activity for at least 1 s before the stimulation was applied. The final score was presented as the integral of the rectified EMG response normalized to baseline, with a magnitude of 0 indicating that no response was calculated above the baseline. The evoked reflex was divided into the short polysynaptic reflex (SPR; 0 ms – 40 ms), longer polysynaptic reflex (LPR; 40 ms – 500 ms), the long-lasting reflex (LLR; subdivided into LLR1, 500 ms – 1.5 s, LLR2, 1.5 s – 2.5 s, and LLR 3, 2.5 s – 3.5 s), and the total long-lasting reflex (total LLR, 500 ms – 3.5 s), and the total signal (summed trace; 40 ms – 3.5 s) were calculated.

#### 2.4.2 *Ex vivo* sacral cord preparation

Sacral cord preparation was conducted on injured male WT (*n* = 11), female WT (*n* = 16), male KO (*n* = 6), and female KO (*n* = 7) mice to evaluate spasm-like activity *ex vivo*. The detailed methodology for this experiment has been previously described (Mahrous and Elbasiouny, 2017). Briefly, mice were anesthetized with an intraperitoneal injection of urethane (≥ 0.2 g/100g). A dorsal laminectomy exposed the lower half of the spinal cord, and a transection at L5-L6 enabled the removal of the entire sacrocaudal spinal cord (S1-Co2 segments) along with its attached spinal roots. The extracted tissue was maintained *ex vivo* in oxygenated artificial cerebrospinal fluid (ACSF) at room temperature (~21 °C) and allowed to rest for ~ 1 h before testing. The dorsal roots (DRs) and ventral roots (VRs) on both sides of the cord were mounted on bipolar wire electrodes. DRs were connected to an S88 Grass stimulator (A-M Systems), while VRs were connected to differential amplifiers (WPI, 1000x gain, filtered between 300 Hz and 3 kHz). Trains of stimulation were delivered to the DRs (5 pulses, 0.1 ms pulse width, 25 Hz) at intensities of 2x threshold – the minimal stimulation amplitude required to elicit a detectable VR response. The evoked VR responses were quantified by measuring the area under the curve of the rectified signal. To block synaptic inhibition, strychnine and picrotoxin (STR/PTX) were added to the ACSF, and ~ 15 min were allowed to achieve full drug effects. Following drug application, DR stimulation elicited VR responses that persisted for several seconds (spasm-like activity). To prevent adaptation of spasm-like activity with repeated stimulations (Mahrous et al., 2024), a minimum 45-s period without stimulation was ensured. The spasm-like activity was analyzed within the long-lasting reflex (LLR5; 500 ms – 5.5 s and LLR10; 500 ms – 10.5 s). Only the contralateral responses were taken for analysis, as previous studies have shown that evoked spasm-like activity is greater on the contralateral side than on the ipsilateral side (Lin et al., 2019).

### 2.5 Protein analysis

#### 2.5.1 Tissue preparation

Uninjured male WT (*n* = 4), female WT (*n* = 4), male KO (*n* = 4), and female KO (*n* = 4) mice, as well as injured male WT (*n* = 4), female WT (*n* = 4), male KO (*n* = 4), and female KO (*n* = 4) mice were used for western blot analysis. Spinal cord tissue from the lumbar and sacral section of the cord was extracted following perfusion and separated beneath the ventral root L6. The tissue was immediately placed on ice and prepared for homogenization by sonication with T-PER (Thermo Scientific; Waltham, MA, USA) and Halt Protease Inhibitor Cocktail (Thermo Scientific; Waltham, MA, USA) and centrifuged at 10,000 g for 5-min. Supernatant was separated and aliquoted prior to being stored at −80 °C until ready for use.

#### 2.5.2 Western blot

The protein concentration of each sample was determined using Pierce BCA Protein Assay reagent kit (Thermo Scientific; Waltham, MA, USA) in order to ensure equal protein loading per lane. Sample preparation consisted of combining the lysed and homogenized sample with sample buffer, reducing agent (BioRad; Hercules, CA, USA), and T-PER before heating the sample at 100 °C for 5-min to both denature the proteins and ensure that the proteins migrate based on size alone, as described previously (Anastasio et al., 2010; Li et al., 2004). For each sample, 12 μg of protein extract was separated by 10–15% SDS-PAGE (BioRad; Hercules, CA, USA) and transferred to PVDF membrane (Thermo Scientific; Waltham, MA, USA). Membranes were blocked in 5% non-fat dried milk solution for 1-h at room temperature before overnight incubation at 4°C with primary antibodies against 5-HT_2C_R, 5-HT_2A_R, and GAPDH. Each overnight incubation contained one primary antibody; therefore, probing for all antibodies took a total of 3 days. Following primary antibody incubation, each membrane was washed in TBS-T 3X for 5-min/wash before secondary incubation with the corresponding secondary antibody for 1-h at room temperature. Each blot had nine lanes; the first lane was Precision Plus Protein Dual Color Standard (Catalog #1610374, Bio-Rad; Hercules, CA) to visually assess and confirm the molecular weights of the proteins of interest, lanes 2–5 contained KO mouse tissue from either the lumbar or sacral spinal cord, and lanes 6–9 contained WT mouse tissue from the same spinal cord section. A total of eight blots were analyzed: (1) uninjured female KO (*n* = 4) and WT mice (*n* = 4) (lumbar), (2) injured female KO (*n* = 4) and WT mice (*n* = 4) (lumbar), (3) uninjured male KO (*n* = 4) and WT mice (*n* = 4) (lumbar), and (4) injured male KO (*n* = 4) and WT mice (*n* = 4) (lumbar). The subsequent blots (5 through 8) used the same mice (*n* = 4 for each group) as the first four blots, but the sacral section was assessed instead of the lumbar section. The lane setup for each western blot can be found in Supplementary Table 7. Each blot was probed for 5-HT_2C_R (Catalog #MA5-32717, 1:1,000, Thermo Scientific; Waltham, MA, USA, RRID: AB_2809994) overnight on the first day, followed by goat anti-rabbit (Catalog #31460, 1:1,000, Thermo Scientific; Waltham, MA, USA, RRID: AB_228341) secondary antibody. The chemiluminescence kit (SKU #AC2103, Azure Biosystems; Dublin, CA, USA) was used to visualize and capture the immunoreactive band with the Azure 400 system (AZI400-01, Azure Biosystems; Dublin, CA, USA). The blot was then re-probed overnight on the second day with 5-HT_2A_R (sc-166775, 1:500, Santa Cruz Biotechnology, TX, USA, RRID: AB_2233203) primary antibody, followed by goat anti-mouse (Catalog #A-10668, 1:2,000, Thermo Scientific; Waltham, MA, USA, RRID: AB_2534058) secondary antibody and imaged once more. Finally, on the third day, the blot was re-probed overnight with GAPDH (Catalog #MA5-35235, 1:100,000, Thermo Scientific; Waltham, MA, USA, RRID: AB_2849138), a new goat anti-rabbit secondary antibody was used again, and the blot was imaged a final time. The western blots were analyzed using ImageJ software (Fiji, RRID: SCR_002285) to quantify band intensities. Both 5-HT_2C_R and 5-HT_2A_R were normalized to the GAPDH signal intensity.

### 2.6 Statistical analysis

Statistical analysis was done using GraphPad Prism Software (Version 10.0.1, GraphPad, Boston, MA, RRID: SCR_002798), IBM SPSS (Version 29.0.2.0, IBM Corp., Armonk, NY, RRID: SCR_002865), and R Statistical Software (v4.1.0; R Core Team 2021, RRID: SCR_001905). Before analysis, data normality was assessed using the Shapiro–Wilk test. For DigiGait™ and grip strength data, a two-way ANOVA was used to test the effects of sex and injury. Diagnostic plots were also examined to confirm the assumptions of the two-way ANOVA before performing *post hoc* multiple comparisons using Fisher’s LSD test. For all other comparisons, residual plots, Q-Q plots, and homoscedasticity plots were examined to evaluate assumptions of normality and homoscedasticity. If data met normality (per Shapiro-Wilk) and showed no evidence of violation of homoscedasticity, parametric *t*-tests were performed to compare two independent groups. If normality was violated, nonparametric Mann–Whitney U-tests were performed. Main effects and interactions were calculated to determine the standardized magnitude of sex and genotype differences: Partial Eta Squared (η^2^p) was reported for two-way ANOVA comparisons, Cohen’s *d* for *t*-test comparisons, and the rank-biserial correlation coefficient for Mann-Whitney U tests. The Pearson correlation coefficient was evaluated to assess the linear relationship between either mouse length or mouse width and various gait parameters. Actual difference values were calculated by taking the average value of each group and subtracting one group from the other. Statistical significance is denoted as *p < 0.05, **p < 0.01, ***p < 0.001, ****p < 0.0001.

## 3 Results

### 3.1 Motor function in uninjured mice

#### 3.1.1 KO mice exhibit decreased power and stability during forward locomotion

Previous reports have described conflicting results on the involvement of the 5-HT_2C_R in locomotion of uninjured mice (Heisler et al., 2007; Hill et al., 2011). Here, we used the comprehensive DigiGait™ digital treadmill analysis to identify subtle differences and possible motor impairments in both sexes of WT and KO mice during normal ambulation at a fixed velocity.

**Figure 1:**
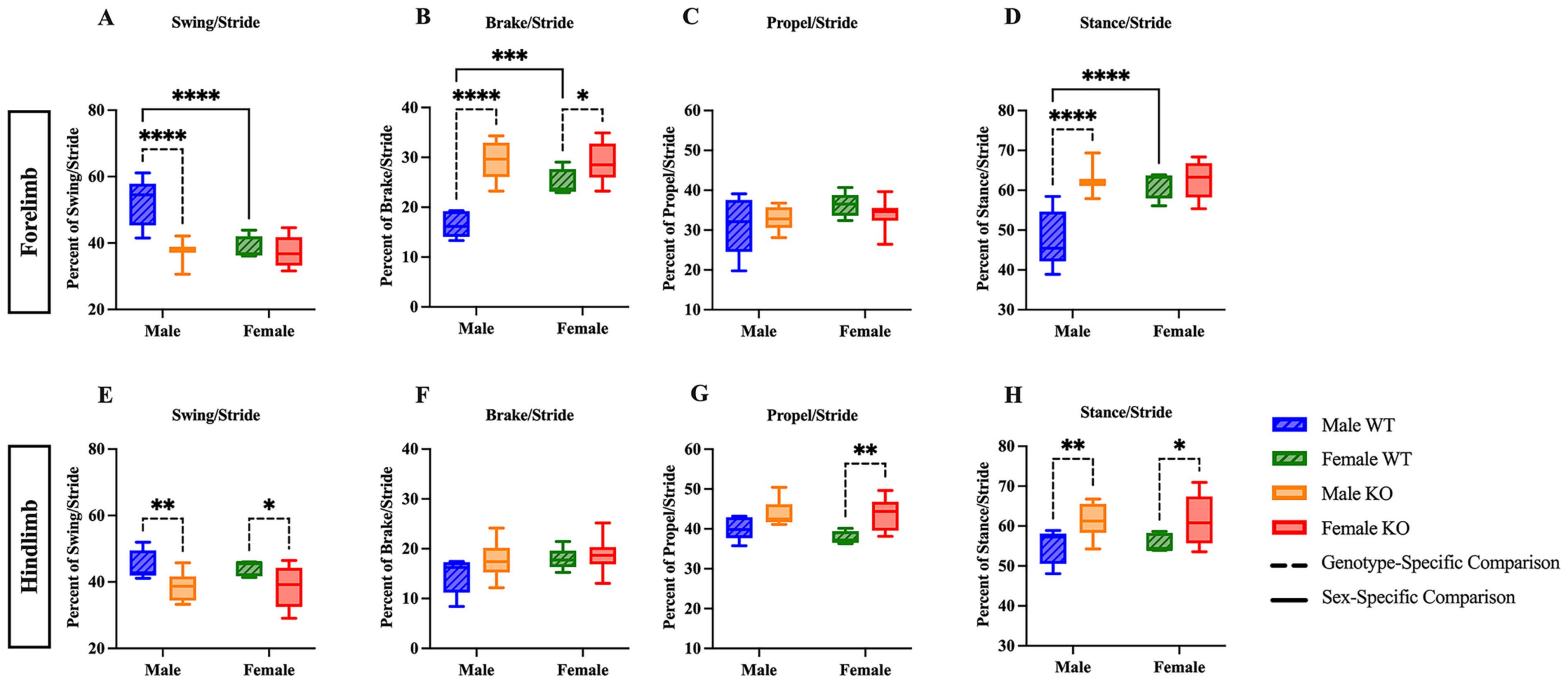
Male and female KO mice show altered stride phase composition compared to their respective WT counterparts. Box plots of the forelimb and hindlimb stride phase composition, including swing **(A,E)**, brake **(B,F)**, propulsion **(C,G)**, and stance (combined brake and propulsion) phases **(D,H)**. Significant differences were assessed using a two-way ANOVA, and *post hoc* comparisons were calculated using Fisher’s LSD test. Male WT (*n* = 5), female WT (*n* = 5), male KO (*n* = 12), and female KO (*n* = 11) mice were included in all analyses. The upper panels show forelimb data; the lower panels show hindlimb data. Solid significance lines indicate sex-specific differences, and dashed significance lines indicate genotype-specific differences. ****p < 0.0001, ***p < 0.001, **p < 0.01, *p < 0.05.

The stride phase consists of the swing and stance phases, with stance further divided into brake (heel contact) and propulsion (toe-off). To assess potential differences in stride composition, the percentage of time spent in swing, brake, propel, and stance (combined brake and propel) phases was analyzed in both the forelimbs and hindlimbs. A two-way ANOVA of the forelimb swing data revealed significant main effects of sex (*F* (1, 29) = 19.04, *p* = 0.0001, η^2^p = 0.396), genotype (*F* (1, 29) = 24.14, *p* < 0.0001, η^2^p = 0.455), and their interaction (*F* (1, 29) = 16.33, *p* = 0.0004, η^2^p = 0.360). *Post hoc* comparisons using Fisher’s LSD revealed that female WT mice spent 13.5 percentage points less time in swing, and male KO mice spent 14.4 percentage points less time in swing compared to male WT mice (Figure 1A) (*p* < 0.0001 for both comparisons). Correspondingly, female WT spent 8.5 percentage points more time in brake, and male KO mice spent 12.9 percentage points more time in brake compared to male WT mice (Figure 1B) (*p* = 0.0006 and *p* < 0.0001, respectively). Female KO mice also spent 4.2 percentage points more time in the forelimb brake phase of stride than female WT mice (*p* = 0.0323). The forelimb data for brake also revealed significant effects of sex (*F* (1, 29) = 9.966, *p* = 0.0037, η^2^p = 0.256), genotype (*F* (1, 29) = 41.82, *p* < 0.0001, η^2^p = 0.591), and their interaction (*F* (1, 29) = 10.66, *p* = 0.0028, η^2^p = 0.269). The hindlimb swing two-way ANOVA revealed a significant main effect of genotype (*F* (1, 29) = 13.63, *p* = 0.0009, η^2^p = 0.320), but not sex nor their interaction. Fisher’s LSD *post hoc* comparisons indicate genotype-specific differences, with male KO mice spending 6.9 percentage points less time in hindlimb swing, and female KO mice spending 6.1 percentage points less time in hindlimb swing than their WT counterparts (Figure 1E) (*p* = 0.0096 and *p = 0*.0206, respectively); however, no differences were revealed in the hindlimb analysis of brake (Figure 1F). While the percentage of time spent in the forelimb propel phase during stride remained unchanged across groups (Figure 1C), the hindlimb propel phase was significantly greater in female KO mice than female WT mice by 5.5 percentage points (Figure 1G) (*p* = 0.0048), and the two-way ANOVA revealed a significant main effect of genotype (*F* (1, 29) = 13.03, *p* = 0.0011, η^2^p = 0.310). Differences in the forelimb stance phase were sex- and genotype-dependent (Figure 1D), with female WT spending 13.5 percentage points more time of the forelimb stride in stance, and male KO mice spending 14.4 percentage points more time of the forelimb stride in stance compared to male WT mice (*p* < 0.0001 for both comparisons). Additionally, the two-way ANOVA for hindlimb stance revealed a significant effect of genotype (*F* (1, 29) = 13.63, *p* = 0.0009, η^2^p = 0.320), and post hoc comparisons indicate that both male and female KO mice spent a greater percentage (6.9 percentage points and 6.1 percentage points, respectively) of hindlimb stride in stance compared to their WT counterparts (*p* = 0.0096 and *p* = 0.0206, respectively (Figure 1H). These results suggest mild sex- and genotype-specific differences in gait stride composition between male and female WT and KO mice.

**Figure 2:**
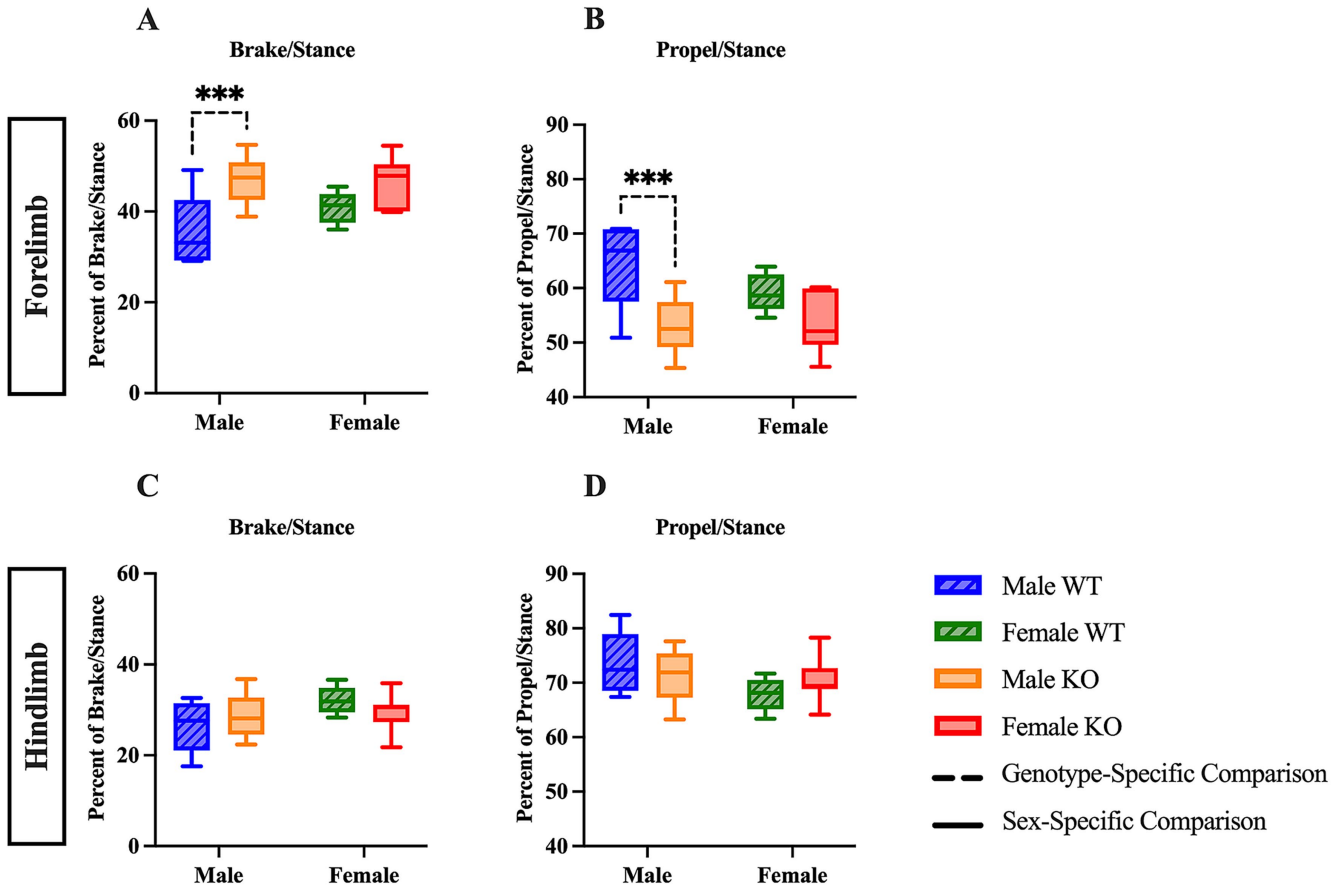
Male KO mice show altered stance phase composition compared to male WT mice. Box plots of stance phase composition, including brake **(A,C)** and propulsion **(B,D)** phases, in the forelimbs and hindlimbs of male and female WT and KO mice. Significant differences were assessed using a two-way ANOVA, and post hoc comparisons were calculated using Fisher’s LSD test. Male WT (*n* = 5), female WT (*n* = 5), male KO (*n* = 12), and female KO (*n* = 11) mice were included in all analyses. The upper panels show forelimb data; the lower panels show hindlimb data. Solid significance lines indicate sex-specific differences, and dashed significance lines indicate genotype-specific differences. ****p < 0.0001, ***p < 0.001, **p < 0.01, *p < 0.05.

As mentioned above, the stance phase can be further divided into brake and propel to provide additional information concerning the time at which either the heel or walking pad of the paw is contacting the belt. Upon independent examination of the stance phase, a two-way ANOVA of the forelimb brake and propel analyses revealed a significant main effect of genotype (*F* (1, 29) = 13.63, *p* = 0.0009, η^2^p = 0.390). *Post hoc* comparisons of the forelimb stance phase indicate that male KO mice spent 11.8 percentage points more time in brake (Figure 2A) (*p* = 0.0003), and 11.8 percentage points less time in propel (Figure 2B) (*p* = 0.0003) than male WT mice. In the hindlimb stance phase analyses, no differences were revealed across groups (Figures 2C,D). These results suggest that male KO mice have pronounced phenotypic alterations in forelimb stance composition, as the amount of time spent with the heel on the belt was greater in male KO mice than in male WT mice, and the amount of time spent with the walking pad of the paw on the belt was less in male KO mice than in male WT mice.

**Figure 3:**
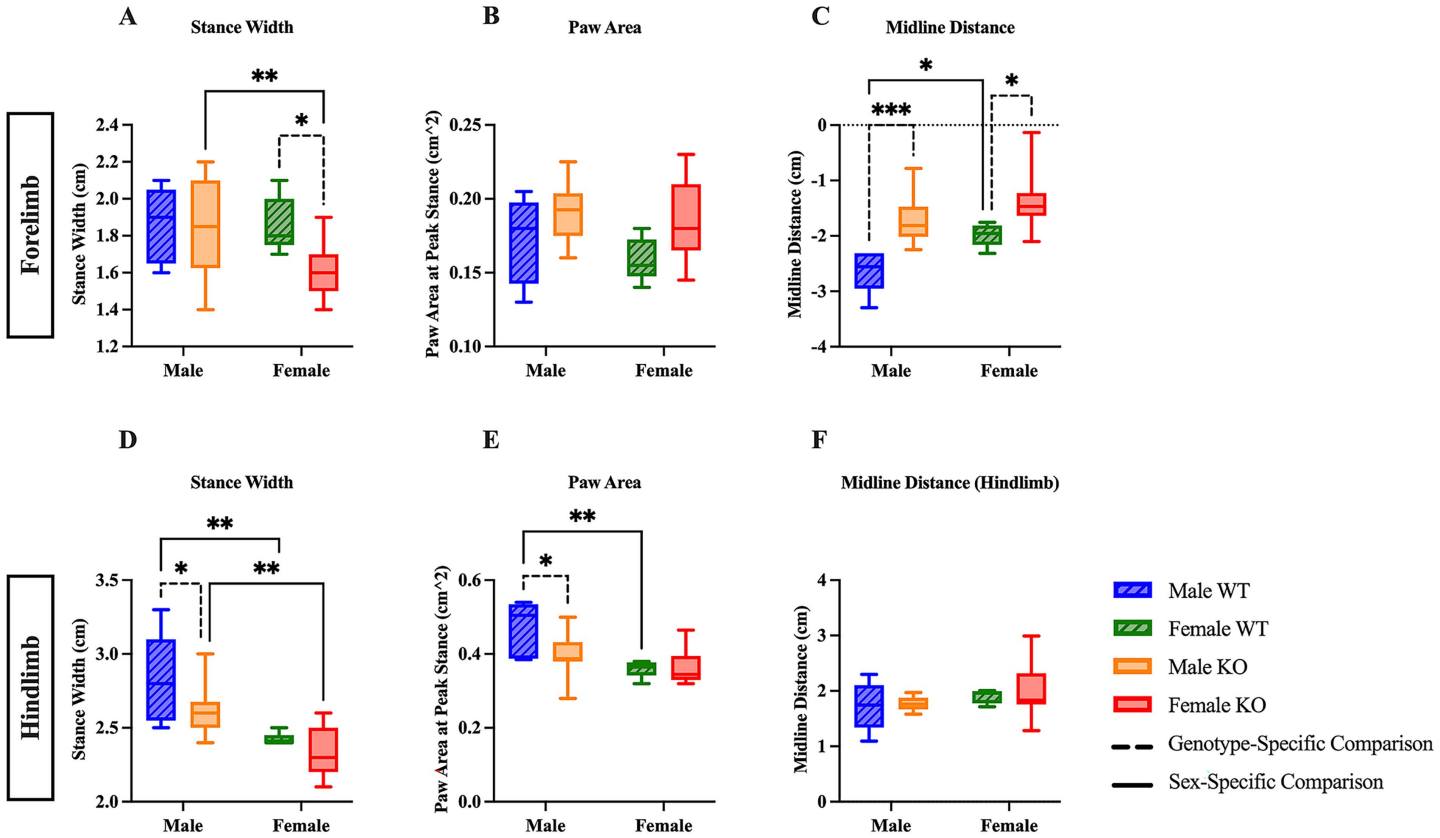
Sex- and genotype-specific differences in stance width, paw area, and midline distance. Box plots of stance width **(A,D)**, paw area **(B,E)**, and midline distance **(C,F)** in the forelimbs and hindlimbs of male and female WT and KO mice. Significant differences were assessed using a two-way ANOVA, and post hoc comparisons were calculated using Fisher’s LSD test. Male WT (*n* = 5), female WT (*n* = 5), male KO (*n* = 12), and female KO (*n* = 11) mice were included in all analyses. The upper panels show forelimb data; the lower panels show hindlimb data. Solid significance lines indicate sex-specific differences, and dashed significance lines indicate genotype-specific differences. ****p < 0.0001, ***p < 0.001, **p < 0.01, *p < 0.05.

The stance width, the measured distance between either the forepaws or the hindpaws, of male and female WT and KO mice was compared to illuminate potential differences in the spatial arrangement of the forelimbs and hindlimbs. Sex-specific differences in stance width were revealed in the *post hoc* comparisons of the two-way ANOVA; however, only hindlimb stance width revealed significant main effects of sex (*F* (1, 29) = 23.37, *p* < 0.0001, η^2^p = 0.446) and genotype (*F* (1, 29) = 0.1703, *p* = 0.0324, η^2^p = 0.148) analyses. Post hoc comparisons show that female KO mice had a narrower stance width in the forelimbs by 0.23 cm (Figure 3A) (*p* = 0.0096) and hindlimbs by 0.27 cm (Figure 3D) (*p* = 0.0013) compared to male KO mice, whereas only the hindlimb stance width of female WT mice was more narrow than male WT mice by 0.4 cm (Figure 3D) (*p* = 0.0018). Genotype-specific differences varied between forelimb and hindlimb analyses, as female KO mice had a narrower forelimb stance width than female WT mice by 0.23 cm (Figure 3A) (*p* = 0.0390) and male KO mice had a narrower hindlimb stance width than male WT mice by 0.22 cm (Figure 3D) (*p* = 0.0321). Notably, these results suggest an inherent sex-specific difference in hindlimb stance width between male and female mice, regardless of whether the 5-HT_2C_R is functional or not, in addition to mild genotype-specific differences between WT and KO mice.

We next examined the paw area, or the surface area of the forepaw and hindpaw during peak stance, in which the largest surface area of the paw is in contact with the belt, to uncover differences between WT and KO mice relating to the overall paw contact and toe splay during locomotion. Interestingly, the two-way ANOVA of forepaw area revealed a significant main effect of genotype (*F* (1, 29) = 5.630, *p* = 0.0245, η^2^p = 0.163), whereas the two-way ANOVA of hindpaw area revealed a significant main effect of sex (*F* (1, 29) = 12.60, *p* = 0.0013, η^2^p = 0.303). There were no differences in forelimb paw area between male or female WT and KO mice (Figure 3B). The hindlimb paw area of female WT was smaller than male WT mice by 0.11 cm^2^, and the hindlimb paw area of male KO mice was also smaller than male WT mice by 0.07 cm^2^ (Figure 3E) (*p* = 0.0029 and *p* = 0.0165, respectively). These results suggest that the absence of the functional 5-HT_2C_R likely affects the paw surface area of male, but not female KO mice, and that there may be a sex-specific biological difference in paw area between male and female WT mice.

Lastly, midline distance, the measurement of the total distance from the midline of the mouse’s body to its forelimb or hindlimb paw, was assessed to evaluate differences in paw positioning relative to the midline, which can reflect the mouse’s relative stability and posturing during locomotion. In the forelimb analysis, a two-way ANOVA revealed significant main effects of sex (*F* (1, 29) = 8.255, *p* = 0.0075, η^2^p = 0.222) and genotype (*F* (1, 29) = 20.18, *p* = 0.0001, η^2^p = 0.410). *Post hoc* comparisons reveal that male and female KO mice had less distance (0.91 cm and 0.63 cm, respectively) between the transverse midline and the forelimb paw than their respective WT counterparts (Figure 3C) (*p* = 0.0007 and *p* = 0.0154, respectively). Note that the negative midline distance on the *y*-axis is due to the directionality of the measurement. Additionally, female WT mice had less distance between the transverse midline and the forelimb paw by 0.64 cm than male WT mice (Figure 3C) (*p* = 0.0346). There were no differences in hindlimb midline distance between any group of mice (Figure 3H). These results suggest both inherent biological differences in WT mice, as well as the possibility that the KO mice lack the necessary muscular power and strength to extend the forelimbs to the extent that the WT mice are capable of, potentially indicating worsened stability during locomotion.

After assessing all gait parameters, we investigated whether the size of the mouse had any significant effect on stride length, stance width, and midline distance – all parameters that could be influenced by a longer or wider mouse. Notably, both WT and KO mice of both sexes had significant correlations with various parameters. There was a significant positive correlation between mouse length and the forelimb stance width of male WT mice (*r* (3) = 0.90, *p* = 0.0386), forelimb and hindlimb midline distance of male WT mice (*r* (3) = 0.91, *p* = 0.0297 and *r* (3) = 0.98, *p* = 0.0032, respectively), and the forelimb midline distance of female KO mice (*r* (9) = 0.81, *p* = 0.0023) were significantly correlated with mouse length. There was a significant positive correlation between mouse width and the hindlimb stance width of male WT mice (*r* (3) = 0.99, *p* = 0.0004), the hindlimb stance width of male and female KO mice (*r* (10) = 0.73, *p* = 0.0073 and *r* (9) = 0.70, *p* = 0.0157, respectively), and the forelimb midline distance of male WT mice (*r* (3) = 0.95, *p* = 0.0143). These results suggest that the individual mouse length and width may have a significant effect on specific gait parameters assessed using the DigiGait™ system, regardless of whether the 5-HT_2C_R is functional or not.

#### 3.1.2 Female KO mice exhibit reduced hindlimb and all-limb grip strength

**Figure 4:**
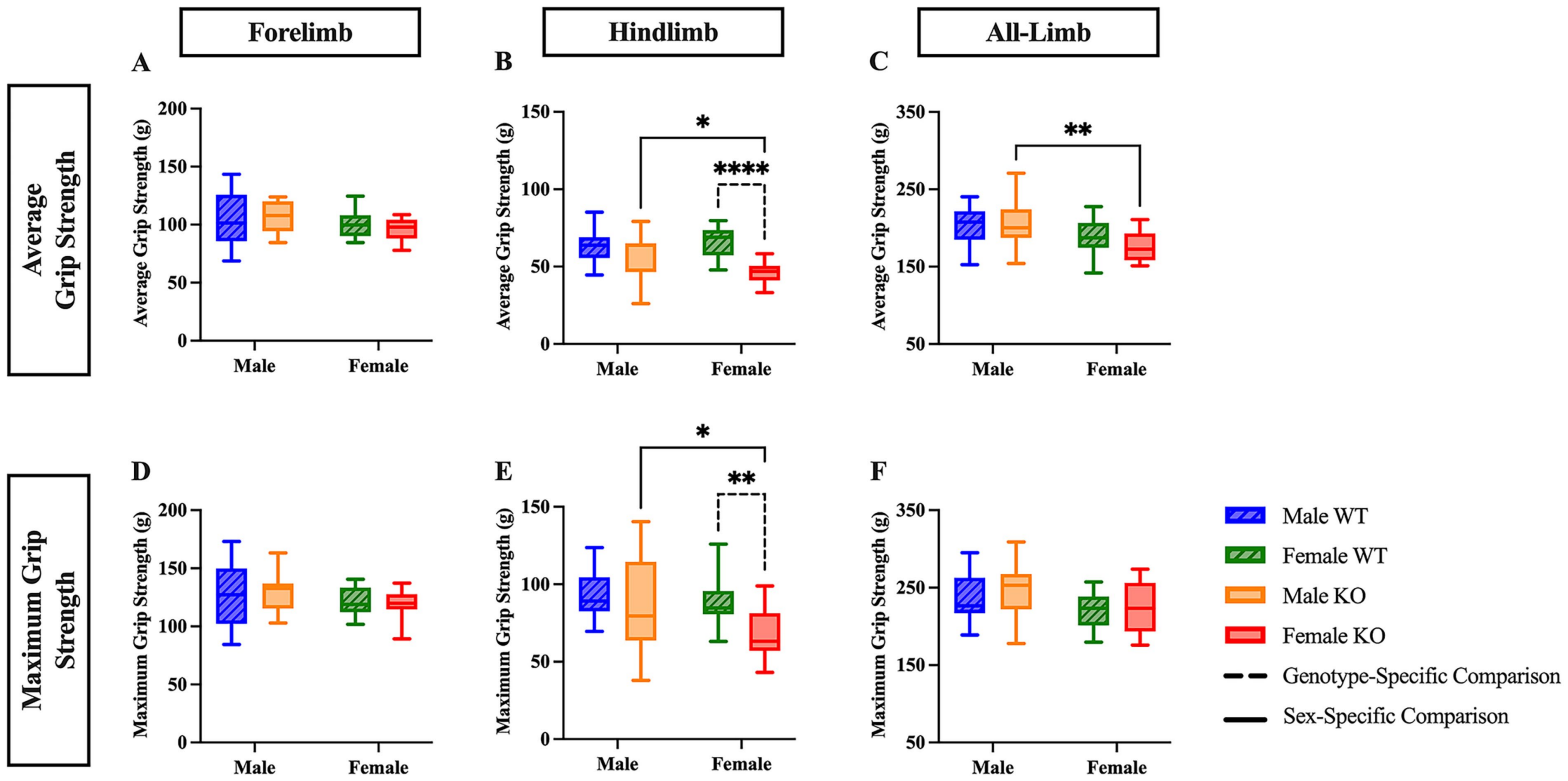
Female KO mice exhibit reduced hindlimb and all-limb grip strength compared to female WT and male KO mice. Box plots of forelimb **(A,D)**, hindlimb **(B,E)**, and all-limb **(C,F)** average and maximum grip strength in male and female WT and KO mice. Significant differences were assessed using a two-way ANOVA, and post hoc comparisons were calculated using Fisher’s LSD test. Male WT (*n* = 18), female WT (*n* = 19), male KO (*n* = 12), and female KO (*n* = 12) mice were included in all analyses. The upper panels show average grip strength data; the lower panels show maximum grip strength data. Solid significance lines indicate sex-specific differences, and dashed significance lines indicate genotype-specific differences. ****p < 0.0001, ***p < 0.001, **p < 0.01, *p < 0.05.

The average grip strength was next assessed in the forelimbs, hindlimbs, and in a combined all-limb measurement, as volitional ambulation is largely influenced by muscular strength. The peak force generated during a single, maximum-effort trial was also recorded (maximum grip strength). There were no significant differences revealed in the forelimb analyses (Figure 4A and 4D). In the hindlimb average analysis, a two-way ANOVA revealed significant main effects of both genotype (*F* (1, 57) = 22.73, *p* < 0.0001, η^2^p = 0.260) and the interaction between sex and genotype (*F* (1, 57) = 7.742, *p* = 0.0073, η^2^p = 0.120). In the hindlimb maximum analysis, a two-way ANOVA revealed significant main effects of both genotype (*F* (1, 57) = 8.954, *p* = 0.0041, η^2^p = 0.136) and sex (*F* (1, 57) = 4.456, *p* = 0.0392, η^2^p = 0.072). *Post hoc* comparisons revealed that female KO mice showed diminished average hindlimb grip strength by 20.4 g (Figure 4B) (*p* < 0.0001) and diminished maximum hindlimb grip strength by 21 g (Figure 4E) (*p* = 0.0043) compared to female WT mice, suggesting that female KO mice exhibit hindlimb muscle weakness compared to their WT counterparts. Additionally, female KO mice had lower hindlimb average grip strength by 11.1 g (Figure 4B) (*p* = 0.0131), lower hindlimb maximum grip strength by 16.6 g (Figure 4E) (*p* = 0.0385), and lower all-limb average grip strength by 29.8g (Figure 4C) (*p* = 0.0039) than male KO mice, suggesting a biological, sex-specific phenomenon exclusive to the mice that lacked the functional 5-HT_2C_R. For the all-limb analyses, a two-way ANOVA revealed significant main effects of sex in both average (*F* (1, 57) = 11.95, *p* = 0.0010, η^2^p = 0.173) and maximum (*F* (1, 57) = 6.474, *p* = 0.0137, η^2^p = 0.102) all-limb grip strength. There were no significant differences revealed in all-limb maximum grip strength between any group (Figure 4F).

### 3.2 Motor function post-SCI

#### 3.2.1 KO mice exhibited less spasm-like activity *ex vivo* post-SCI compared to WT mice

**Figure 5:**
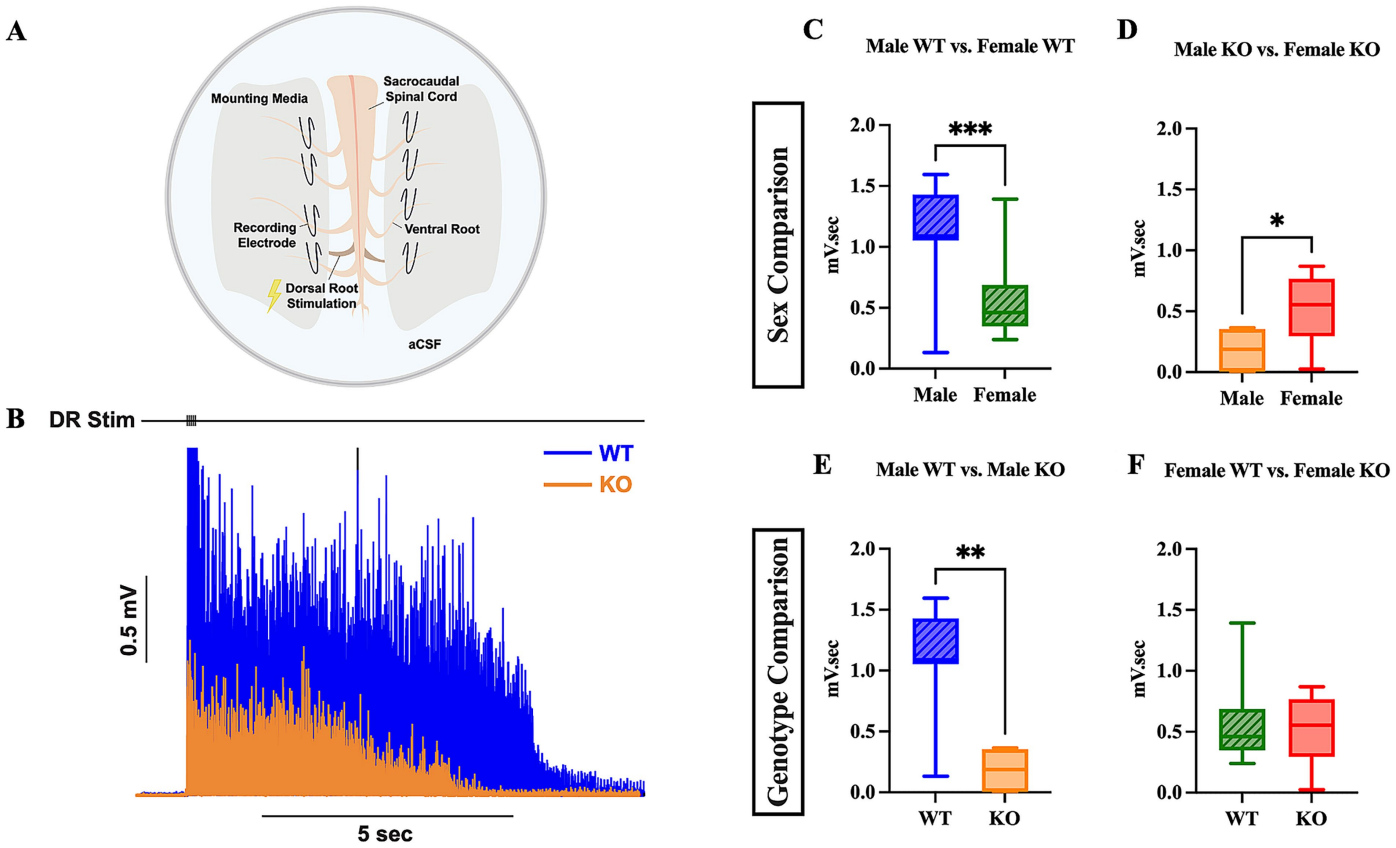
Male KO and female WT mice exhibit reduced spasm-like activity compared to male WT mice. Visual schematic of sacral cord preparation setup **(A)** and representative LLR ENG trace from sacral root over 10 s **(B)**. Box plots compared LLR (10 s) activity between male WT and female WT **(C)**, male KO and female KO **(D)**, male WT and male KO **(E)**, and female WT and female KO **(F)** mice. An unpaired *t*-test was used for comparison between male KO and female KO mice; all other comparisons used a Mann–Whitney test due to non-normal data distributions. Male WT (*n* = 11), female WT (*n* = 16), male KO (*n* = 6), and female KO (*n* = 7) mice were included in all analyses. ****p < 0.0001, ***p < 0.001, **p < 0.01, *p < 0.05.

The reduction in hyperreflexia observed in 5-HT_2C_R KO mice may be due to a decrease in MN PICs, which are known to be facilitated by this specific receptor (Murray et al., 2010; Tysseling et al., 2017). We employed *ex vivo* ENG recording to further assess the long-latency response elicited from the ventral root in both SCI WT and KO mice, as previous research has shown that low-threshold stimulation evokes long-lasting reflexes (Bennett et al., 2004; Mahrous et al., 2024). We used 2x threshold stimulation of the dorsal roots on either side of the cord to activate low threshold and high threshold afferents, respectively (Figure 5A). The evoked ventral root long-latency response (LLR) over a period of 10 s was recorded and used for analysis (Figure 5B). Female WT and male KO mice had diminished LLR by 0.56 mV.sec and 0.92 mV.sec, respectively, than male WT mice (Figures 5C,E) (*p* = 0.0010 and *p* = 0.0011, respectively). Male KO mice also had diminished LLR compared to female KO mice by 0.30 mV.sec (Figure 5D) (*p* = 0.0496). There were no significant differences in LLR revealed between female WT and female KO mice (Figure 5F). These results, which were corroborated by similar findings in the flexor withdrawal reflex analysis (see below), suggest that the male KO mice exclusively exhibit decreased spasm-like activity (or motor output) compared to male WT mice. The results of the WT comparison also suggest a difference between sex, in which female WT mice have less spasm-like activity than male WT mice with a large effect size of sex (Mann-Whitney U-test, rank-biserial correlation coefficient *r* = 0.61). These results may indicate that the sex of the mouse and the 5-HT_2C_R both play a prominent role in enhancing spasms in male mice that have undergone chronic SCI, potentially via the increased facilitation of MN PICs.

#### 3.2.2 Both sex- and genotype-specific differences revealed in western blot protein analysis

All three of the 5-HT_2_ receptor subtypes have been shown to possess similar molecular structure, pharmacology, and signal transduction pathways to one another (Parajulee and Kim, 2023). Of the three 5-HT_2_ receptor subtypes, 5-HT_2A_ and 5-HT_2C_ have been the most extensively studied for their role in motor function. It is unclear as to whether the 5-HT_2C_R or 5-HT_2A_R is upregulated post-SCI, because previous research using total protein analysis and mRNA analysis has presented conflicting results. Previous total mRNA studies examining rats that have undergone sacral transection have shown that the 5-HT_2C_R is not upregulated after sacral transection, but the constitutive activity of the receptors that are present is upregulated (Murray et al., 2010). Conversely, immunohistochemical studies and total protein analyses of the 5-HT_2C_R have shown that the 5-HT_2C_R protein is upregulated in the sacral cord 60 days post-sacral spinal transection as compared to sham rats (Murray et al., 2010; Ren et al., 2013). In research conducted to investigate the closely related 5-HT_2A_R, it has been suggested that the 5-HT_2A_R is upregulated in the sacral spinal cord post-sacral spinal transection (Kong et al., 2010).

**Figure 6:**
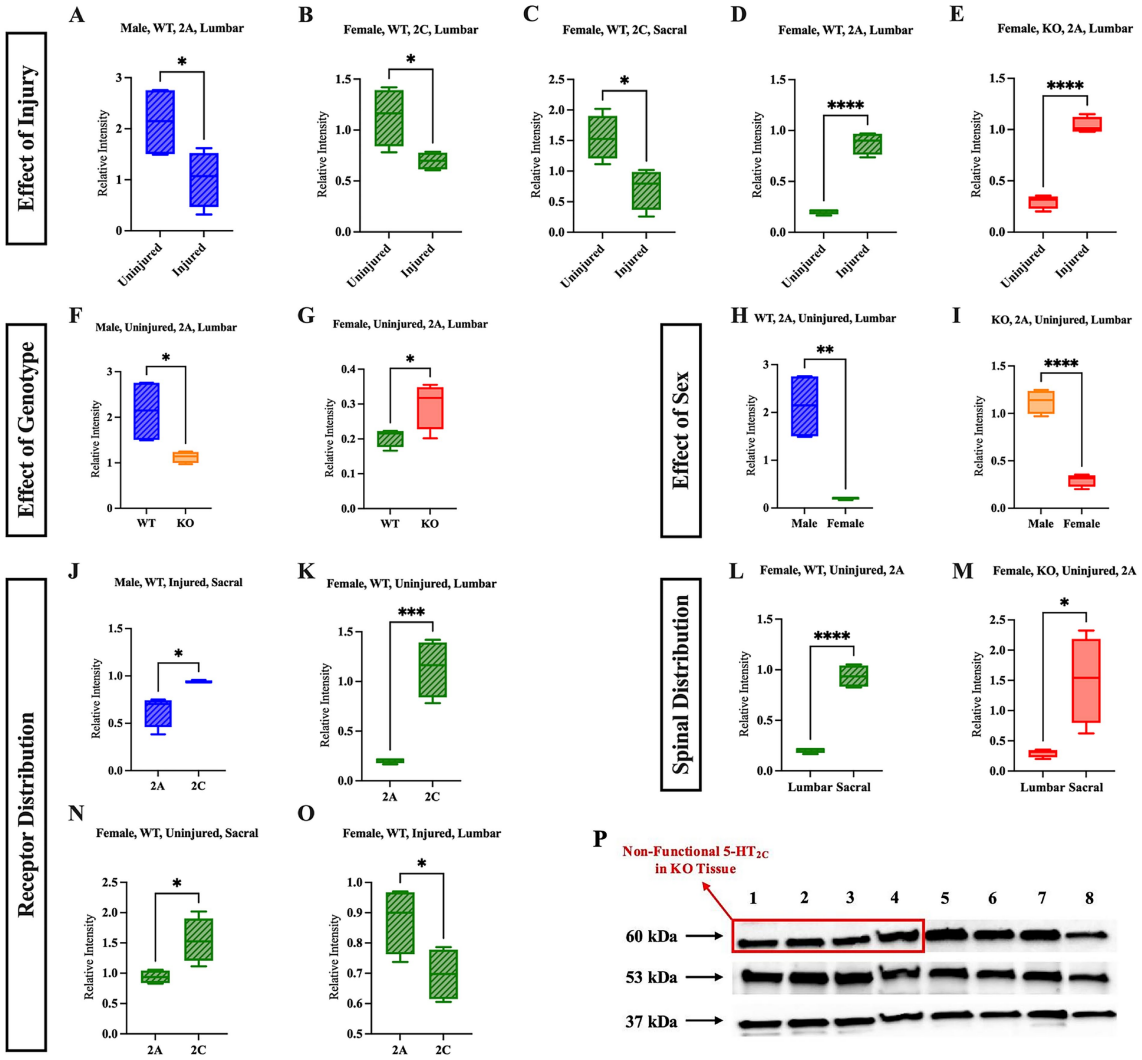
Sex, genotype, and injury status affect the relative expression and distribution of 5-HT_2A_R and 5-HT_2C_R. The effect of injury is shown in panels **(A–E)**; the effect of genotype (WT vs. KO) is shown in panels **(F,G)**; the effect of sex (male vs. female) is shown in panels **(H,I)**; the relative receptor distribution (5-HT_2A_R vs. 5-HT_2C_R) is shown in **(J,K,N,O)**; and the spinal distribution of a specific receptor (lumbar vs. sacral) is shown in panels **(L,M)**. A representative western blot **(P)** in which lanes 1–4 are four different tissue samples from the lumbar cord of uninjured female KO mice, and lanes 5–8 are four different tissue samples from the lumbar uninjured female WT mice. Significant differences were assessed using an unpaired *t-*test Uninjured male WT (*n* = 4), uninjured female WT (*n* = 4), uninjured male KO (*n* = 4), uninjured female KO (*n* = 4), injured male WT (*n* = 4), injured female WT (*n* = 4), injured male KO (*n* = 4), injured female KO (*n* = 4) mice were included in all analyses. ****p < 0.0001, ***p < 0.001, **p < 0.01, *p < 0.05.

To further examine possible mechanistic alterations that occur in WT and KO mice post-SCI, western blotting of spinal cord tissue was conducted to quantify the relative expression of 5-HT_2C_R and 5-HT_2A_R in both the lumbar and sacral spinal cord of both uninjured and injured WT and 5-HT_2C_R KO mice. A representative western blot showing 5-HT_2C_, 5-HT_2A_, and GAPDH expression is presented, with lanes 1–4 containing lumbar tissue extracted from four different uninjured female KO mice and lanes 5–8 containing lumbar tissue extracted from four different uninjured female WT mice, is shown (Figure 6P). Full western blot images are shown in Supplementary Figures 7–10. It is critical to address the presence of the 5-HT_2C_ band in the uninjured female KO mice [as seen in the red rectangular box (Figure 6P)]. The foundational paper in which the specific 5-HT_2C_R KO mouse model was generated explicitly states that a nonsense mutation was introduced into exon 5 of the cognate gene, thereby eliminating the carboxy-terminal half of the protein. To the best of our knowledge, there is no monoclonal primary antibody available for use in which the antibody binds to the c-terminus of the 5-HT_2C_R protein, and the reactive species is suitable for the mouse. Ultimately, it is well understood and confirmed with Jackson Laboratories that the 5-HT_2C_R is non-functional in the KO model used in this study, and the 5-HT_2C_R bands observed in the male and female KO mice were excluded and not considered for analysis because of the non-functionality of the receptor.

The following five comparisons were investigated: effect of injury on relative receptor quantity within each group, effect of genotype on relative receptor quantity within each sex, effect of sex on relative receptor quantity within each genotype, relative receptor distribution within the spinal section (lumbar or sacral), and relative receptor distribution across spinal sections. All parametric western blot results can be found in Supplementary Table 5, and all non-parametric western blot results can be found in Supplementary Table 6.

Five significant results were revealed when assessing the effect of SCI on the relative quantity of receptors. A full list of non-significant results can be found in Supplementary Tables 1, 2. Notably, four of these injury-related results were revealed in WT mice, while only one was revealed in KO mice. The relative quantity of the 5-HT_2C_R decreased after injury in the lumbar region and decreased in the sacral region of female WT mice (Figures 6B,C) (*p* = 0.0281 and *p* = 0.0158, respectively). In the lumbar region, the relative quantity of 5-HT_2A_R decreased after injury in male WT mice (Figure 6A) (*p* = 0.0486). In contrast to these results, the relative quantity of 5-HT_2A_R increased after injury in the lumbar cord of female WT mice (Figure 6D) (*p* < 0.0001) and increased in the lumbar cord of female KO mice after injury (Figure 6E) (*p* < 0.0001).

There were two significant differences revealed when the genotype was compared. While uninjured male KO mice had relatively less 5-HT_2A_R expression in the lumbar section than uninjured male WT mice (Figure 6F) (*p* = 0.0322), uninjured female KO mice had relatively greater expression of 5-HT_2A_R in the lumbar section than uninjured female WT mice (Figure 6G) (*p* = 0.0422). These findings suggest that uninjured male and female KO mice have different baseline relative expression of 5-HT_2A_R relative to WT mice of their respective sex in the lumbar, but not sacral, section of the spinal cord.

Sex comparisons revealed two significant differences as well. In the lumbar section of both uninjured female WT and female KO mice, the relative expression of 5-HT_2A_R was reduced compared to their respective male counterparts of the same genotype (Figures 6H,I) (*p* = 0.0017 and *p* < 0.0001, respectively).

There were numerous differences revealed upon comparing the quantities of the two receptors to one another. This analysis increases our understanding of how the expression of each receptor differed within spinal sections, sex, and genotype. Injured male WT mice had greater 5-HT_2C_R than 5-HT_2A_R in the sacral cord (Figure 6J) (*p* = 0.0124), and uninjured female WT mice had greater 5-HT_2C_R than 5-HT_2A_R in both the lumbar and sacral cord (Figures 6K,N) (*p* = 0.0007 and *p* = 0.0428, respectively). Interestingly, female WT mice had more 5-HT_2A_R than 5-HT_2C_R in the sacral cord after injury (Figure 6O) (*p* = 0.0007).

The receptor distribution across spinal sections was also evaluated to identify differences between the relative quantity of 5-HT_2C_R and 5-HT_2A_R within the lumbar and sacral spinal cord of each mouse group. In both uninjured female WT and KO mice, 5-HT_2A_ expression was greater in the sacral cord compared to the lumbar cord (Figures 6L,M) (*p* < 0.0001 and *p* = 0.0155, respectively). This result aligns with the previous findings, in which it was suggested that the female mice may be characterized by an intrinsic homeostatic mechanism that upregulates the 5-HT_2A_R post-SCI.

#### 3.2.3 Male KO mice exhibited less hyperreflexia than male WT mice post-SCI

**Figure 7:**
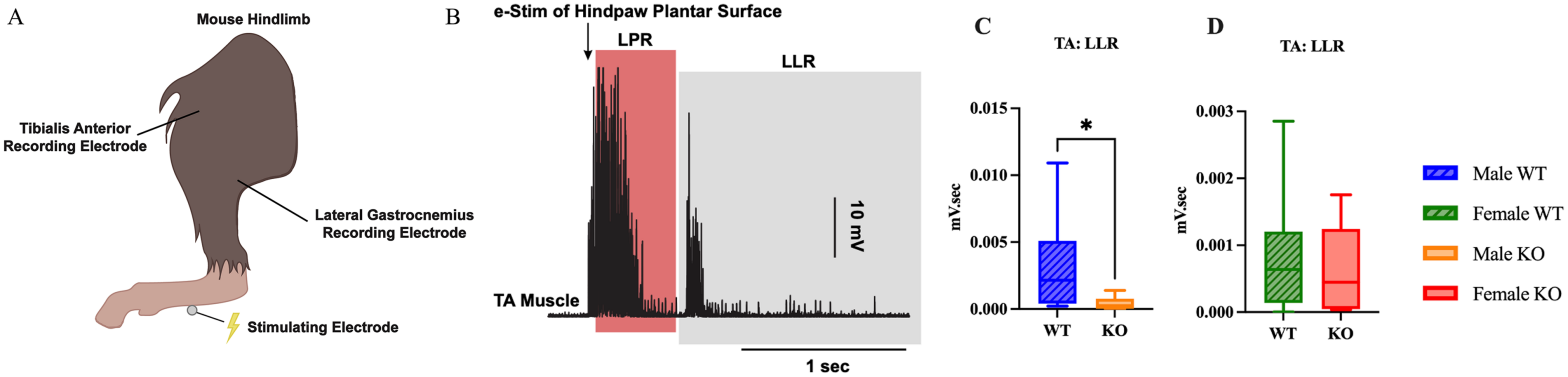
Male KO mice exhibit reduced hyperreflexia compared to male WT mice. Visual schematic of the flexor withdrawal stimulating electrode and recording electrode placement in the mouse hindlimb **(A)**, and representative male WT and male KO EMG traces with labeled LLR and LPR labeled **(B)**. Box plots illustrating genotype-specific differences in LLR of the TA muscle between male WT and KO mice **(C)**, and female WT and KO mice **(D)**. Male WT (*n* = 8), male KO (*n* = 8), female WT (*n* = 11), and female KO (*n* = 9) mice were included in the analyses. A Mann–Whitney test was used for comparison between male WT and KO mice due to non-normal data distributions; an unpaired *t*-test was used for comparison between female WT and KO mice. LLR, long-latency response; LPR, longer polysynaptic response. ****p < 0.0001, ***p < 0.001, **p < 0.01, *p < 0.05.

To assess whether the 5-HT_2C_R KO impacts the presence or extent of hyperreflexia following SCI, the flexor withdrawal reflex response was assessed *in vivo* by electrically stimulating the plantar side of the hindpaw while recording EMG activity in the LG and TA antagonistic muscle pair (Figures 7A,B). There was no difference in LG activity between either sex or genotype (Supplementary Figures 2, 3). In the TA, however, male KO mice had 0.0029 mV.sec less LLR EMG activity than male WT mice (Figure 7C) (*p* = 0.0379, Mann–Whitney U-test, *r* = 0.53). However, there were no significant differences in LLR revealed between female WT and female KO mice in the TA (Figure 7D). This suggests that the loss of the functional 5-HT_2C_R resulted in significantly reduced hyperreflexia in male mice, as revealed through decreased EMG activity from the TA muscle post-SCI. All flexor withdrawal reflex response results for the TA muscle can be found in Supplementary Figures 4, 5.

#### 3.2.4 No difference in gross motor recovery post-SCI between KO and WT mice

The BMS open-field behavioral assessment was conducted to assess gross motor recovery near the chronic stage of injury. No differences were revealed between either sex or genotype of the mice (Supplementary Figure 6), suggesting that the recovery between male and female mice, regardless of genotype, did not differ. By week 10, no mouse scored above a 2, which is characterized by extensive ankle movement but no coordinated or consistent stepping activity, suggesting the transections were complete and that no mice used in the study regained gross motor movement post-SCI.

## 4 Discussion

This study was conducted to achieve a more comprehensive understanding of how the 5-HT_2C_R and sex differences are implicated in volitional and involuntary motor behavior in uninjured mice and post-SCI. In uninjured mice, the absence of the functional 5-HT_2C_R was shown to be associated with mild changes in gait kinematics and muscular strength. In SCI mice, the absence of the functional 5-HT_2C_R was shown to be associated with decreased involuntary motor behaviors post-SCI, which was corroborated by the relative protein expression analyses. The protein analyses showed that the 5-HT_2C_R is primarily influenced by injury status, whereas the 5-HT_2A_R is more broadly influenced by the sex, genotype, and injury status of the mouse.

### 4.1 In uninjured mice, the 5-HT_2C_R marginally contributes to strength and stability during volitional movement

#### 4.1.1 Genotype-specific differences in volitional locomotion between uninjured WT and KO mice

Comparing typical WT mice to a 5-HT_2C_R KO model by dissecting locomotion into specific gait kinematics using DigiGait™ is a novel approach used to achieve a greater understanding of whether the KO mice exhibit altered gait characteristics and stability deficits that have been previously unaccounted for in broader analyses of volitional behavior, such as open-field assessments.

Male KO mice spent 14.4 percentage points less time in forelimb, 6.9 percentage points less time in hindlimb swing (a single-legged position), and more time in double-legged positions, such as forelimb brake (12.9 percentage points), and both forelimb (14.4 percentage points) and hindlimb (6.9 percentage points) stance compared to male WT mice. It is important to note that the combined percentage of time spent in swing and stance is 100% because that is a full gait cycle. Therefore, male KO mice spend less time in swing and an equivalent percentage more time in stance. Female KO mice also spent 6.1 percentage points less time in hindlimb swing and 6.1 percentage points more time in hindlimb stance than female WT mice, suggesting that the KO mice exhibit mild proximal weakness and possibly compensate by increasing the amount of time spent in more-stable, double-legged positions as compared to male WT mice.

Male KO mice were also characterized as having a narrower hindlimb stance width by 0.22 cm and shorter forelimb midline distance by 0.91 cm compared to male WT mice, whereas female KO mice had a narrower forelimb stance width by 0.23 cm as well as a shorter midline distance by 0.63 cm compared to female WT mice. These findings suggest that the KO mice have a weaker foundational base of stability, as indicated by the tendency to keep the forelimbs near the midline and the hindlimb stance width closer than the WT mice of the respective sex. These differences in gait kinematics may suggest that the KO mice are unable to sustain stable positioning during locomotion. Recent studies conducted in aged mice have shown that the 5-HT_2C_ agonist, Lorcaserin, significantly increased cervical motor evoked potential (cMEP) amplitude and improved both motor output and grip strength in mice (Kerr et al., 2025). Our findings provide further support that the 5-HT_2C_R is directly implicated in muscle contraction and volitional movement, as the loss of the functional 5-HT_2C_R results in marginal decreases in both muscular strength and stability in male mice.

#### 4.1.2 Sex-specific differences in volitional locomotion between uninjured male and female mice

In addition to genotype-specific differences, comparisons between sexes within each genotype were assessed to ensure that potential innate biological differences between male and female mice were also addressed – a key difference between the current study and previous research. Emerging literature has shown significant differences between young adult (aged 18–40 years old) male and female human subjects regarding numerous gait parameters, including dynamic stability (Al-Makhalas et al., 2023). Notably, the study from Al-Makhalas et al. suggests that antero-posterior stability was greater in male than female subjects, a finding that is supported by the current study.

Female WT mice spent 13.5 percentage points less time in forelimb swing and 8.5 percentage points more time in forelimb brake and forelimb stance (13.5 percentage points) than male WT mice. These findings align with the genotype-specific results, in which male KO mice displayed mild proximal weakness and reduced stability as compared to male WT mice. This similarity between male KO mice and female WT mice may explain the lack of sex-specific differences between male and female KO mice. Furthermore, female KO mice exhibited decreased forelimb stance width by 0.23 cm compared to male KO mice, and both female WT and KO mice exhibited reduced hindlimb stance width (by 0.4 cm and 0.27 cm, respectively) compared to the male mice of their respective genotype. Female WT mice had a smaller paw area by 0.11 cm^2^ and shorter midline distance by 0.64 cm than male WT mice – once again replicating the same significant differences as those observed in the male KO mice. The presence of sex-specific significant differences suggests an inherent biological difference between sexes, with female mice exhibiting a more unstable gait during locomotion compared to male mice.

The average body length and width of each group (i.e., male WT, male KO, female WT, and female KO) of mice, and the correlation that those measurements had with the gait parameters, stride length, stance width, and midline distance were evaluated. It was hypothesized that larger mice would have different gait kinematics compared to smaller mice, which may be a sex-dependent phenomenon. Notably, the length of male WT mice had a significant correlation with forelimb stance width and forelimb and hindlimb midline distance. The width of male WT mice had a significant correlation with hindlimb stance width and forelimb midline distance. The length of female KO mice also had a significant correlation with forelimb midline distance, and the width of male and female KO mice also had a significant correlation with hindlimb stance width. These results suggest that the body size of each group of mice must not be ignored as a potential contributing factor to various gait parameters. It has been previously established that male mice have greater overall muscle mass as compared to female mice (Oydanich et al., 2019), which may explain the strong correlation with overall mouse length and width. Interestingly, the KO mice reflect similar correlations as well, suggesting that these sex differences are inherent regardless of the functional 5-HT_2C_R.

#### 4.1.3 Genotype-specific differences in volitional grip strength between WT and KO mice

Although previous research has focused on investigating locomotive ability regarding 5-HT_2C_R, serotonin projection within the spinal cord has also been shown to regulate muscle tone (Stamm et al., 2017). This research has suggested a strong positive relationship between muscle tone (tonic motor activity) and the firing rate of 5-HT neurons in the dorsal raphe nucleus, which has projections to the spinal cord (Jacobs et al., 2002; Zhang et al., 2024). It is largely unknown whether the 5-HT_2C_R is directly implicated in muscle tone; however, previous research has shown that the 5-HT_2_R does regulate motor unit discharge rates and contributes to voluntary muscle contraction in human subjects (Goodlich et al., 2023). Our results further support the connection between grip strength and the 5-HT_2C_R. Female KO mice had weaker hindlimb average grip strength by 20.4 g and maximum hindlimb grip strength by 21 g compared to female WT mice, suggesting a marginal genotype-specific difference in distal muscular strength that predominantly appears in female KO mice, and is not observed in male KO mice.

#### 4.1.4 Sex-specific differences in volitional grip strength between male and female mice

Female KO mice exhibited weaker average and maximum hindlimb grip strength by 11.1 g and 16.6 g, respectively, as well as weaker average all-limb grip strength than male KO mice by 29.8 g, suggesting that the female KO mice exhibit a mild, distal weakness in all limbs that is sex-dependent and is not replicated in the WT mice. Female KO mice also exhibit weaker average and hindlimb grip strength compared to female WT mice, but this finding is not replicated in the male mice. As mentioned previously, it is well established that in the vast majority of mammalian lineages, males tend to have greater muscle mass than females – i.e., sexual dimorphism is present regarding this characteristic (Oydanich et al., 2019). This could contribute to the sex-specific differences observed in the female KO mice. Previous studies have identified two specific genes that are upregulated in females compared to males, growth factor receptor-bound protein 10 (GRB10) and activin receptor type-2A (ACVR2A), both of which are implicated in the regulation of skeletal muscle mass. However, the difference in muscle composition between sexes is thought to entail many more signaling pathways and genes than just the two identified thus far (Haizlip et al., 2015). Future studies assessing sex-specific differences in both volitional grip strength and gait kinematics between male and female mice, especially those that aim to understand the function of a specific gene, should strongly consider using *ex vivo* and *in vivo* experimental techniques to compare muscle fiber composition between both sexes and genotype to better understand sex differences in volitional grip strength.

In human studies, previous research has shown that administering the 5-HT_2_ antagonist cyproheptadine reduced cMEP amplitude during voluntary contraction of the biceps brachii during elbow flexion and at rest (Henderson et al., 2024; Thorstensen et al., 2021). These previous studies, in combination with the present study, provide strong evidence that 5-HT_2_ influences motoneuron excitability and motor output in both human and mouse subjects. These influences are particularly evident in assessments of volitional motor behavior, such as voluntary locomotion and grip strength.

### 4.2 Following SCI, KO mice exhibit less involuntary motor behavior *ex vivo* than WT mice

Our study utilized an SCI mouse model to evaluate potential sex- and genotype-specific differences in involuntary motor behavior after injury. We hypothesized that the injured KO mice would exhibit decreased involuntary motor behaviors compared to the WT mice because 5-HT_2C_R signaling below the level of injury has been previously suggested to contribute to MN PIC activity and hyperreflexia in SCI mice (Murray et al., 2011a; Tysseling et al., 2017).

#### 4.2.1 Genotype-specific differences in *ex vivo* hyperreflexia between WT and KO mice

*Ex vivo* sacral cord preparation was used to assess hyperreflexia and spasm-like activity in the spinal cords of SCI mice. No differences in hyperreflexia were revealed between female KO mice and their WT counterparts. However, male KO mice had significantly less hyperreflexia by 0.92 mV.sec compared to male WT mice. This result supports not only our hypothesis, but it also provides additional evidence in support of previous research that has shown that 5-HT_2C_R signaling below the level of injury contributes to MN PIC activity and hyperreflexia post-SCI.

Previous research using female rats has shown that the administration of the antagonist SB206553, which is used to block the 5-HT_2C_R and 5-HT_2B_R, effectively inhibits the 5-HT_2_ agonist-induced increase in LLR, suggesting that these two specific receptors facilitate the LLR phase of spasms (Murray et al., 2011a). Although no differences were revealed between female WT and female KO mice in LLR10, the LLR5 (ranging from 500 ms post-stimulation to 5,500 ms) data revealed that the median value was greater in female KO mice (median = 0.4301, *n* = 16) than female WT (median = 0.3833, *n* = 7) mice (Supplementary Figure 1). A five-second LLR is much closer to the paper cited above, in which the LLR was quantified by averaging the response that ranged from 500 ms post-stimulation to 4,000 ms. Additionally, the antagonist SB206553 blocks both the 5-HT_2C_R *and* the 5-HT_2B_R, the latter of which was unaccounted for in our analyses. Although the 5-HT_2C_R was genetically knocked out, we did not account for possible upregulation or other homeostatic mechanisms that may affect the expression of the 5-HT_2B_R. Additional research is necessary to distinguish between the specific roles of both the 5-HT_2B_R and 5-HT_2C_R to determine how spasm-like activity is mediated by each receptor.

Furthermore, it is critical to acknowledge that the 5-HT_2C_ gene is X-linked, meaning that females have two copies of this gene, whereas male mice only have one copy (Heisler and Tecott, 2000). To the best of our knowledge, it remains unknown whether the second copy of the gene is silenced in female mice, as is typical of X-linked genes through the process of X-chromosome inactivation (Carrel and Brown, 2017; Lyon, 1961). Additional research, inclusive of heterozygous female KO mice, is required to fully understand whether the double copy of the 5-HT_2C_ gene causes additional effects on female (XX) mice, as it is unknown whether this gene is fully silenced and ineffective or if there are indeed pronounced alterations in volitional (i.e., gait and grip strength) and involuntary (i.e., *in vivo* flexor withdrawal and *ex vivo* sacral cord preparation) motor behaviors due to the presence of a double gene.

#### 4.2.2 Sex-specific differences in *ex vivo* hyperreflexia between male and female mice

The *ex vivo* data also revealed that female WT mice had significantly less hyperreflexia by 0.56 mV.sec than male WT mice. This may suggest a sex-dependent phenomenon or a differing homeostatic mechanism between male and female WT mice. Previous research has shown evidence that female WT rats that have undergone an incomplete SCI (iSCI) have greater spontaneous locomotor recovery than male WT rats (Hauben et al., 2002). Subsequent studies have shown that T cells involved in neuroprotection from secondary mechanisms of iSCI are specifically characterized by a sex-dependent phenomenon (Hauben et al., 2002, 2001; Moalem et al., 1999). Our findings provide additional evidence for a sex-dependent phenomenon regarding involuntary motor behavior (hyperreflexia) following complete SCI. In contrast to the WT data, female KO mice had greater hyperreflexia compared to male KO mice. However, this result may reflect a basement effect, as ENG activity was extremely reduced in the male KO mice.

### 4.3 Sex, genotype, and injury-dependent modulation of 5-HT_2C_R and 5-HT_2A_R expression in the lumbar and sacral spinal cord

As 5-HT_2C_R and 5-HT_2A_R have both been suggested to be involved in motor function, the goal of quantifying the relative protein expression of the 5-HT_2C_R and 5-HT_2A_R was to further examine how SCI, sex, and 5-HT_2C_R KO influence the quantity and distribution of each receptor in the lumbar and sacral spinal cord. Understanding potential changes in serotonergic signaling and receptor quantity may illuminate or provide supplementary evidence for the differences observed in volitional motor behavior in uninjured mice, and in involuntary motor behavior post-SCI.

#### 4.3.1 Effect of injury on 5-HT_2C_R and 5-HT_2A_R protein expression

Our results suggest that the relative expression of 5-HT_2C_R decreased post-SCI in the lumbar and sacral spinal cord of female WT mice. Previous research has shown no difference in overall 5-HT_2C_R mRNA expression post-SCI; however, the amount of RNA editing at a single site in the 5-HT_2C_R RNA has been shown to decrease after injury, leading to the increased expression of a specific isoform (INI), which facilitates PIC activity (Murray et al., 2010). A possible explanation for this discrepancy is that mRNA expression measures the quantity of transcripts produced from a gene. However, it does not account for certain post-transcriptional or translational regulatory mechanisms that regulate functional protein expression that can be detected via western blot analysis. Our findings suggest the presence of potential changes in 5-HT_2C_R translational regulatory mechanisms or receptor degradation.

The relative quantity of the 5-HT_2A_R decreased after injury in the lumbar cord of male WT mice, but increased in the lumbar cord of female WT and female KO mice post-SCI. This upregulation in female KO mice may reflect a compensatory response to the absence of the functional 5-HT_2C_R. Correspondingly, *ex vivo* data show greater ENG activity in female KO mice compared to male KO mice, as well as a greater, though non-significant, median ENG activity than in female WT mice. These findings further support the presence of compensatory mechanisms involving 5-HT_2A_R expression in female KO mice. Although female WT mice also exhibit increased 5-HT_2A_R (and a decrease in 5-HT_2C_R) post-SCI, this upregulation does not translate into heightened motoneuron excitability in the *ex vivo* data. This suggests that the degree of 5-HT_2A_R compensation in female WT mice is insufficient to match the functional compensation observed in female KO mice.

In regard to the decrease in 5-HT_2A_R in the lumbar cord of male WT mice, previous research has shown that the immunoreactivity of the 5-HT_2A_R in male spinalized rats is upregulated below the lesion site compared to male sham rats (Kong et al., 2010). Contrarily, another study provided evidence that expression of 5-HT and the 5-HT transporter was downregulated after 2 days post-SCI, suggesting that the 5-HT_2A_R may have a role in exacerbating involuntary motor behaviors (Kong et al., 2011). It is necessary to consider that the previous study visualized the S4 and Ca1 segments of the spinal cord after spinalization at the S2 level, whereas the current study used whole-tissue western blotting of the lumbar and sacral spinal cord following SCI transection at the T10 level, and all significant results were revealed in the lumbar cord, not the sacral. Notably, differences in 5-HT_2A_R expression between the present study and Kong et al. may be attributed to secondary injury effects such as ischemia and inflammation, which are known to impact regions of the spinal cord beyond the lesion site (Anjum et al., 2020).

#### 4.3.2 Effect of sex on 5-HT_2C_R and 5-HT_2A_R protein expression

In uninjured mice, both female WT and KO mice had less relative expression of the 5-HT_2A_R than their respective male counterparts in the lumbar spinal cord. It would be negligent to dismiss the well-established link between serotonin and sex hormones upon discussing sex differences between male and female mice. Previous studies have shown an inconclusive involvement of the estradiol-17β (E_2_) receptor in serotonin binding and synthesis (Bendis et al., 2024). Some studies have shown that E_2_ stimulates an increase in 5-HT_2A_R expression in the dorsal raphe nucleus of female rats (Sumner and Fink, 1995), which may have a downstream effect on 5-HT within the spinal cord, stimulating increased 5-HT_2A_R protein expression in the lumbar or sacral cord. Interestingly, our western blot results show that this is not the case in the spinal cord; they instead suggest that the effect of sex gives rise to a more complicated picture. However, it is unknown whether there are underlying homeostatic mechanisms within the reticulospinal tract that would alter the 5-HT_2A_R quantity in the lumbar or sacral spinal cord, citing a need for further research on the relationship between E_2_ receptors and 5-HT within the spinal cord itself.

Furthermore, it has been widely established that the E_2_ receptor fluctuates both daily and monthly in females (Bendis et al., 2024). The menstrual cycle of female mice was not tracked through this study. The absence of this tracking is a confounding variable because the potential fluctuations in sex hormones that have been previously shown to impact the serotonergic system were not accounted for in our analyses. The menstrual cycle of female mice may have a significant impact on 5-HT_2C_R and/or 5-HT_2A_R expression, directly impacting the results of our western blot analyses and possibly extending further to affect the motor behavior results as well. Future studies investigating the effect of serotonergic receptors on male and female mouse behavior and tissue must consider tracking the menstrual cycle of female mice to provide further clarity on hormonal alterations that may directly impact their results.

#### 4.3.3 Effect of 5-HT_2C_R KO mutation on 5-HT_2A_R protein expression

As discussed previously, the 5-HT_2C_R and 5-HT_2A_R are similar in structure, signaling, and function, as they are both subtypes of the 5-HT_2_ receptor family and are both involved in motor control (Fonseca et al., 2001; Halberstadt et al., 2009; Parajulee and Kim, 2023). Due to these similarities, it was hypothesized that the absence of the functional 5-HT_2C_R in the KO mice would cause an upregulation of the 5-HT_2A_R through a compensatory molecular mechanism. This hypothesis was only supported in uninjured female KO mice. Uninjured female KO mice had greater 5-HT_2A_R expression in the lumbar spinal cord than uninjured female WT mice, but uninjured male KO mice had lower 5-HT_2A_R expression in the lumbar spinal cord compared to uninjured male WT mice. These findings suggest a sex-dependent homeostatic mechanism possibly linking the regulation of the 5-HT_2A_R to the relative expression of the 5-HT_2C_R within the lumbar and sacral spinal cord, but additional research is necessary to fully understand the connection between 5-HT_2C_R and 5-HT_2A_R.

### 4.4 In SCI mice, male KO mice exhibit decreased involuntary motor behavior *in vivo*

#### 4.4.1 Genotype-specific differences in *in vivo* hyperreflexia between WT and KO mice

An *in vivo* assessment was employed to examine the flexor withdrawal response reflex in awake, SCI mice of each group. While sex- and genotype-specific differences were revealed in the *ex vivo* analysis, the *in vivo* analysis revealed only genotype-specific differences. There was a significant decrease in hyperreflexia by 0.0029 mV.sec observed in the LLR of the TA muscle in the male KO mice compared to the male WT mice.

One explanation for this data is that female mice, having no difference in the amount of hyperreflexia when compared with male mice, may have a different internal mechanism connected to MN excitability and PICs after SCI. Another possible explanation for why the *in vivo* and *ex vivo* LLR data differ between sexes may be related to regional specificity within the spinal cord. *Ex vivo* sacral cord preparation involves stimulation and recording of the sacral spinal roots, whereas *in vivo* flexor withdrawal testing involves stimulation of sensory input to the lumbar spinal cord, and the motor output is recorded in the hindlimb muscle.

No differences in any phase of the response (i.e., LPR, LLR, TS) were revealed in the LG muscle. The TA and LG form an antagonist muscle pair and work in opposition to one another during the flexor withdrawal reflex. Following stimulation, the TA is stretched while the LG shortens due to the positioning of the limb and direction of the reflexive movement. This joint angle may preferentially engage the TA, contributing to the significant differences observed in this specific muscle, whereas the LG may have a more passive role and thereby limited EMG activity. There may also be a basement effect observed, as the overall EMG activity in the LG is much lower than the TA.

## 5 Future directions and limitations

The findings presented in the current study suggest that the 5-HT_2C_ receptor may serve as a novel therapeutic target to reduce involuntary motor behavior post-SCI. Future studies are necessary to better understand the specific mechanistic alterations that occur in the corticospinal circuitry following SCI, and how these alterations specifically affect the 5-HT_2C_ receptor. Only the 5-HT_2C_R and 5-HT_2A_R were investigated in the present study; however, previous research has shown that both the 5-HT_2B_R and other noradrenergic inputs may also possess underlying mechanistic compensatory changes (Kiehn et al., 1999; Lee and Heckman, 1999, p. 25; Murray et al., 2011a; Sqalli-Houssaini and Cazalets, 2000). Without fully understanding these additional inputs, the role the 5-HT_2C_R has in motor function and involuntary motor behaviors post-SCI may be underestimated. Future research is needed to address the possible combinatorial relationship between noradrenergic, dopaminergic, and serotonergic input in spinal cord circuitry, both in uninjured and SCI subjects.

The current study is not without limitations. In the volitional assessments, the body weight of each mouse used was not recorded. Therefore, grip strength data were not normalized to body weight. This is a limitation in that the data collected from the grip strength assessment may reflect differences in mouse body weight, not true functional strength differences. Future studies assessing physiological strength should strongly consider normalization to subject weight to increase confidence in reporting true muscular strength differences.

Furthermore, previous human research has emerged showing that the initial firing rate of motor units in the vastus medialis and the vastus medialis oblique during an isometric ramp knee extension in female subjects fluctuates across the menstrual cycle (Tenan et al., 2013). The current study did not track the menstrual cycle of female mice; however, as sex differences were revealed in multiple experiments conducted, the lack of menstrual tracking may be a confounding variable if motor unit discharge varied significantly between female subjects. Future studies should strongly consider tracking the menstrual cycle of female mice when assessing sex differences between male and female mice.

A prominent statistical limitation is the small sample size used in the western blot analyses. Only four biological replicates per group, across sixteen experimental conditions, were used in this experiment. This limited number of replicates reduces the statistical power to detect subtle differences, as well as increases the sensitivity to outliers or variability between replicates. The differences revealed in our data reached significance, suggesting that the significant effects are unlikely to be due to random variation. Regardless, increasing the sample size of mice used in the western blot analysis would improve both the statistical confidence and effect estimates. Future studies are strongly encouraged to increase the sample size of biological replicates to validate the findings from the current study and assess the generalizability of the results presented.

Importantly, the current study does not address potential compensatory changes in neuromodulation that arise from the birth of the 5-HT_2C_R KO mice. Due to the absence of the functional 5-HT_2C_R from birth, other 5-HT_2_ receptors, especially those that are similar in molecular structure and signal transduction pathways (Barnes and Sharp, 1999), may compensate to adapt for any possible deficits observed in the 5-HT_2C_R KO mice. Therefore, it is challenging to definitively link the 5-HT_2C_R to the outcomes of this study without further analysis on both the dopaminergic and noradrenergic systems, as well as the other 5-HT_2_ receptors.

## 6 Conclusion

This study provides comprehensive behavioral and protein expression analyses that examine how the absence of the functional 5-HT_2C_R can affect volitional and involuntary motor behavior, and how the expression of two specific 5-HT receptors previously shown to be involved in motor function are altered in the sacral and lumbar spinal cord post-SCI. This study examined two serotonergic receptors from the 5-HT_2_ family, the 5-HT_2A_R and 5-HT_2C_R, and their possible effect on MN excitability post-SCI. However, serotonergic input is not the only input that is capable of mediating MN excitability post-SCI. The noradrenaline receptor, for example, has been shown to facilitate the recovery of PICs following a SCI (Heckman et al., 2009). Future research might consider additional analysis tailored to compare serotonergic receptors and noradrenergic receptors in order to determine whether one source of input has a larger effect on involuntary motor behavior post-SCI.

Gaining a comprehensive understanding of how the 5-HT_2C_R is involved in both volitional and involuntary movement post-SCI is necessary in order to determine how these receptors contribute to locomotive recovery and/or the severity of hyperreflexia/spasms. Examining how the expression and quantity of the 5-HT_2C_R and 5-HT_2A_R are altered in the lumbar and sacral spinal cord post-SCI in both male and female mice may provide further insight that would allow for the development of novel and precise therapeutic strategies that target these specific receptors to assist in reducing the frequency and severity of muscle spasms post-SCI.

## Data Availability

The raw data supporting the conclusions of this article will be made available by the authors, without undue reservation.

## Supplementary material

SUPPLEMENTARY FIGURE 1 Male KO and female WT mice exhibit reduced spasm-like activity in LLR 5 (s) compared to male WT mice. Box plots compared LLR (5 s) activity between male and female WT mice **(A)**, male and female KO mice **(B)**, male WT and male KO mice **(C)**, and female WT and female KO mice **(D)**. An unpaired *t*-test was used for comparison between male and female mice; all other comparisons used a Mann-Whitney test due to non-normal data distributions. Male WT (*n* = 11), female WT (*n* = 16), male KO (*n* = 6), and female KO (*n* = 7) mice were included in all analyses. *****p* < 0.0001, ****p* < 0.001, ***p* < 0.01, **p* < 0.05.

SUPPLEMENTARY FIGURE 2 No significant differences in hyperreflexia in the LG muscle between sexes within each genotype. Box plots illustrating sex-specific differences in LPR **(A,D)**, LLR **(B,E)**, and total signal **(C,F)** of the LG muscle between male and female WT mice (upper panel), and male and female KO mice (lower panel). Male WT (*n* = 8), male KO (*n* = 8), female WT (*n* = 11), and female KO (*n* = 9) were included in the analyses. An unpaired *t*-test was used for comparison of LPR and total signal between male and female WT mice and LLR between male and female KO mice; a Mann-Whitney test was used for all other comparisons due to non-normal data distributions. LLR, long-latency response; LPR, longer polysynaptic response. *****p* < 0.0001, ****p* < 0.001, ***p* < 0.01, **p* < 0.05.

SUPPLEMENTARY FIGURE 3 No significant differences in hyperreflexia in the LG muscle between genotypes within each sex. Box plots illustrating genotype-specific differences in LPR **(A,D)**, LLR **(B,E)**, and total signal **(C,F)** of the LG muscle between male WT and KO mice (upper panel), and female WT and KO mice (lower panel). Male WT (*n* = 8), male KO (*n* = 8), female WT (*n* = 11), and female KO (*n* = 9) were included in the analyses. An unpaired *t*-test was used for comparison of LPR between female WT and KO mice and total signal between male WT and KO mice; a Mann-Whitney test was used for all other comparisons due to non-normal data distributions. LLR, long-latency response; LPR, longer polysynaptic response. *****p* < 0.0001, ****p* < 0.001, ***p* < 0.01, **p* < 0.05.

SUPPLEMENTARY FIGURE 4 No difference in hyperreflexia in the TA muscle between sexes within each genotype. Box plots illustrating sex-specific differences in LPR **(A,D)**, LLR **(B,E)**, and total signal **(C,F)** of the TA muscle between male and female WT mice (upper panel), and male and female KO mice (lower panel). Male WT (*n* = 8), male KO (*n* = 8), female WT (*n* = 11), and female KO (*n* = 9) were included in the analyses. An unpaired *t*-test was used for comparison of LPR, LLR, and total signal between male and female KO mice; a Mann-Whitney test was used for all other comparisons due to non-normal data distributions. LLR, long-latency response; LPR, longer polysynaptic response. *****p* < 0.0001, ****p* < 0.001, ***p* < 0.01, **p* < 0.05.

SUPPLEMENTARY FIGURE 5 Only male KO mice exhibit reduced hyperreflexia compared to male WT mice in the LLR of the TA muscle. Box plots illustrating genotype-specific differences in LPR **(A,D)**, LLR **(B,E)**, and total signal **(C,F)** of the TA muscle between male WT and KO mice (upper panel), and female WT and KO mice (lower panel). Male WT (*n* = 8), male KO (*n* = 8), female WT (*n* = 11), and female KO (*n* = 9) were included in the analyses. An unpaired *t*-test was used for comparison of LPR, LLR, and total signal between female WT and KO mice; a Mann-Whitney test was used for all other comparisons due to non-normal data distributions. LLR, long-latency response; LPR, longer polysynaptic response. *****p* < 0.0001, ****p* < 0.001, ***p* < 0.01, **p* < 0.05.

SUPPLEMENTARY FIGURE 6 No significant difference in averaged Basso Mouse Scale (BMS) scores at chronicity (weeks 8–10). Box plots illustrating sex-specific differences in BMS between male and female WT mice **(A)** and male and female KO mice **(B)**, as well as genotype-specific differences between male WT and KO mice **(C)** and female WT and KO mice **(D)**. Male WT (*n* = 8), male KO (*n* = 5), female WT (*n* = 7), and female KO (*n* = 6) were included in the analyses. An unpaired *t*-test was used for comparison of male and female WT mice and female WT and KO mice; a Mann-Whitney test was used for all other comparisons due to non-normal data distributions. *****p* < 0.0001, ****p* < 0.001, ***p* < 0.01, **p* < 0.05.

SUPPLEMENTARY FIGURE 7 Full western blot results of 5-HT_2C_ as shown in Figure 6P. Western blot analysis of 5-HT_2C_ expression in uninjured female KO mice (bands 1 – 4) and uninjured female WT mice (bands 5 – 8).

SUPPLEMENTARY FIGURE 8 Full western blot results of 5-HT_2A_ as shown in Figure 6P. Western blot analysis of 5-HT_2A_ expression in uninjured female KO mice (bands 1 – 4) and uninjured female WT mice (bands 5 – 8).

SUPPLEMENTARY FIGURE 9 Full western blot results of GAPDH as shown in Figure 6P. Western blot analysis of GAPDH expression in uninjured female KO mice (bands 1 – 4) and uninjured female WT mice (bands 5 – 8).

SUPPLEMENTARY FIGURE 10 Full western blot results of Precision Plus Protein Dual Color Standard. The protein ladder was used in all western blots in lane 1 to visually assess and confirm the molecular weight of chemiluminescent bands from the protein of interest. Molecular weights (kDa) are listed on the left-hand side of each marker.

## References

[ref1] Alizadeh A. Dyck S. M. Karimi-Abdolrezaee S. (2019). Traumatic spinal cord injury: an overview of pathophysiology, models and acute injury mechanisms. Front. Neurol. 10:282. doi: 10.3389/fneur.2019.00282, PMID: 30967837 PMC6439316

[ref2] Al-Makhalas A. Abualait T. Ahsan M. Abdulaziz S. Al Muslem W. (2023). A gender based comparison and correlation of spatiotemporal gait parameters and postural stability: gait parameters and postural stability. Acta Biomed. Atenei Parmensis 94:e2023057. doi: 10.23750/abm.v94i2.13602, PMID: 37092642 PMC10210557

[ref3] Anastasio N. C. Lanfranco M. F. Bubar M. J. Seitz P. K. Stutz S. J. McGinnis A. G.. (2010). Serotonin 5-HT_2C_ receptor protein expression is enriched in synaptosomal and post-synaptic compartments of rat cortex. J. Neurochem. 113, 1504–1515. doi: 10.1111/j.1471-4159.2010.06694.x, PMID: 20345755 PMC2917206

[ref4] Anjum A. Yazid M. D. Fauzi Daud M. Idris J. Ng A. M. H. Selvi Naicker A.. (2020). Spinal cord injury: pathophysiology, multimolecular interactions, and underlying recovery mechanisms. Int. J. Mol. Sci. 21:7533. doi: 10.3390/ijms21207533, PMID: 33066029 PMC7589539

[ref5] Barnes N. M. Sharp T. (1999). A review of central 5-HT receptors and their function. Neuropharmacology 38, 1083–1152. doi: 10.1016/S0028-3908(99)00010-6, PMID: 10462127

[ref6] Basso D. M. Fisher L. C. Anderson A. J. Jakeman L. B. Mctigue D. M. Popovich P. G. (2006). Basso mouse scale for locomotion detects differences in recovery after spinal cord injury in five common mouse strains. J. Neurotrauma 23, 635–659. doi: 10.1089/neu.2006.23.635, PMID: 16689667

[ref7] Bayliss D. A. Umemiya M. Berger A. J. (1995). Inhibition of N- and P-type calcium currents and the after-hyperpolarization in rat motoneurones by serotonin. J. Physiol. 485, 635–647. doi: 10.1113/jphysiol.1995.sp020758, PMID: 7562606 PMC1158033

[ref8] Bendis P. C. Zimmerman S. Onisiforou A. Zanos P. Georgiou P. (2024). The impact of estradiol on serotonin, glutamate, and dopamine systems. Front. Neurosci. 18:1348551. doi: 10.3389/fnins.2024.1348551, PMID: 38586193 PMC10998471

[ref9] Bennett D. J. Sanelli L. Cooke C. L. Harvey P. J. Gorassini M. A. (2004). Spastic long-lasting reflexes in the awake rat after sacral spinal cord injury. J. Neurophysiol. 91, 2247–2258. doi: 10.1152/jn.00946.2003, PMID: 15069102

[ref10] Binder M. D. Heckman C. J. Powers R. K. (2011). “The physiological control of motoneuron activity” in Comprehensive Physiology. Hoboken, New Jersey, USA: John Wiley & Sons, Inc. doi: 10.1002/cphy.cp120101

[ref11] Carrel L. Brown C. J. (2017). When the Lyon(ized chromosome) roars: ongoing expression from an inactive X chromosome. Philos. Trans. R. Soc. B 372:20160355. doi: 10.1098/rstb.2016.0355, PMID: 28947654 PMC5627157

[ref12] Coleman W. (2004). Injury severity as primary predictor of outcome in acute spinal cord injury: retrospective results from a large multicenter clinical trial. Spine J. 4, 373–378. doi: 10.1016/j.spinee.2003.12.006, PMID: 15246294

[ref13] Fonseca M. I. Ni Y. G. Dunning D. D. Miledi R. (2001). Distribution of serotonin 2A, 2C and 3 receptor mRNA in spinal cord and medulla oblongata. Mol. Brain Res. 89, 11–19. doi: 10.1016/S0169-328X(01)00049-3, PMID: 11311971

[ref9001] Goodlich B. I. Del Vecchio A. Horan S. A. Kavanagh J. J. (2023). Blockade of 5‐HT2 receptors suppresses motor unit firing and estimates of persistent inward currents during voluntary muscle contraction in humans. The Journal of Physiology. 601, 1121–1138. doi: 10.1113/JP284164, PMID: 36790076

[ref14] Haizlip K. M. Harrison B. C. Leinwand L. A. (2015). Sex-based differences in skeletal muscle kinetics and Fiber-type composition. Physiology 30, 30–39. doi: 10.1152/physiol.00024.2014, PMID: 25559153 PMC4285578

[ref15] Halberstadt A. L. Van Der Heijden I. Ruderman M. A. Risbrough V. B. Gingrich J. A. Geyer M. A.. (2009). 5-HT2A and 5-HT2C receptors exert opposing effects on locomotor activity in mice. Neuropsychopharmacol 34, 1958–1967. doi: 10.1038/npp.2009.29, PMID: 19322172 PMC2697271

[ref16] Han Q. Ordaz J. D. Liu N.-K. Richardson Z. Wu W. Xia Y.. (2019). Descending motor circuitry required for NT-3 mediated locomotor recovery after spinal cord injury in mice. Nat. Commun. 10:5815. doi: 10.1038/s41467-019-13854-3, PMID: 31862889 PMC6925225

[ref17] Hauben E. Agranov E. Gothilf A. Nevo U. Cohen A. Smirnov I.. (2001). Posttraumatic therapeutic vaccination with modified myelin self-antigen prevents complete paralysis while avoiding autoimmune disease. J. Clin. Invest. 108, 591–599. doi: 10.1172/JCI12837, PMID: 11518733 PMC209402

[ref18] Hauben E. Mizrahi T. Agranov E. Schwartz M. (2002). Sexual dimorphism in the spontaneous recovery from spinal cord injury: a gender gap in beneficial autoimmunity? Eur. J. Neurosci. 16, 1731–1740. doi: 10.1046/j.1460-9568.2002.02241.x, PMID: 12431226

[ref19] Heckman C. J. Gorassini M. A. Bennett D. J. (2005). Persistent inward currents in motoneuron dendrites: implications for motor output. Muscle Nerve 31, 135–156. doi: 10.1002/mus.20261, PMID: 15736297

[ref20] Heckman C. J. Lee R. H. Brownstone R. M. (2003). Hyperexcitable dendrites in motoneurons and their neuromodulatory control during motor behavior. Trends Neurosci. 26, 688–695. doi: 10.1016/j.tins.2003.10.002, PMID: 14624854

[ref21] Heckman C. J. Mottram C. Quinlan K. Theiss R. Schuster J. (2009). Motoneuron excitability: the importance of neuromodulatory inputs. Clin. Neurophysiol. 120, 2040–2054. doi: 10.1016/j.clinph.2009.08.009, PMID: 19783207 PMC7312725

[ref22] Heisler L. K. Tecott L. H. (2000). A paradoxical locomotor response in serotonin 5-HT_2C_ receptor mutant mice. J. Neurosci. 20:RC71. doi: 10.1523/JNEUROSCI.20-08-j0003.2000, PMID: 10751458 PMC6772206

[ref23] Heisler L. K. Zhou L. Bajwa P. Hsu J. Tecott L. H. (2007). Serotonin 5-HT_2C_ receptors regulate anxiety-like behavior. Genes Brain Behav. 6, 491–496. doi: 10.1111/j.1601-183X.2007.00316.x, PMID: 17451451

[ref24] Henderson T. T. Taylor J. L. Thorstensen J. R. Kavanagh J. J. (2024). Excitatory drive to spinal motoneurones is necessary for serotonin to modulate motoneurone excitability via 5-HT_2_ receptors in humans. Eur. J. Neurosci. 59, 17–35. doi: 10.1111/ejn.16190, PMID: 37994250

[ref25] Hill R. A. Murray S. S. Halley P. G. Binder M. D. Martin S. J. Van Den Buuse M. (2011). Brain-derived neurotrophic factor expression is increased in the hippocampus of 5-HT_2C_ receptor knockout mice. Hippocampus 21, 434–445. doi: 10.1002/hipo.20759, PMID: 20087884

[ref26] Hounsgaard J. Hultborn H. Jespersen B. Kiehn O. (1988). Bistability of c-motoneurones in the decerebrate cat and in the acute spinal cat after intravenous 5-hydroxytryptophan. J Physiol. 405, 345–367. doi: 10.1113/jphysiol.1988.sp017336 3267153 PMC1190979

[ref27] Jacobs B. L. Martín-Cora F. J. Fornal C. A. (2002). Activity of medullary serotonergic neurons in freely moving animals. Brain Res. Rev. 40, 45–52. doi: 10.1016/S0165-0173(02)00187-X 12589905

[ref28] Jenz S. T. Beauchamp J. A. Gomes M. M. Negro F. Heckman C. J. Pearcey G. E. P. (2023). Estimates of persistent inward currents in lower limb motoneurons are larger in females than in males. J. Neurophysiol. 129, 1322–1333. doi: 10.1152/jn.00043.2023, PMID: 37096909 PMC10202474

[ref29] Kerr N. R. Dashtmian A. R. Darvishi F. B. Brennan C. D. Ayyagari S. N. Moore P. J.. (2025). 5-HT2C agonism as a neurotherapeutic for sarcopenia: preclinical proof of concept. GeroScience 47, 4075–4092. doi: 10.1007/s11357-025-01519-7, PMID: 39825167 PMC12181463

[ref30] Kiehn O. Sillar K. T. Kjaerulff O. McDearmid J. R. (1999). Effects of noradrenaline on locomotor rhythm–generating networks in the isolated neonatal rat spinal cord. J. Neurophysiol. 82, 741–746. doi: 10.1152/jn.1999.82.2.741, PMID: 10444672

[ref31] Kong X.-Y. Wienecke J. Chen M. Hultborn H. Zhang M. (2011). The time course of serotonin 2A receptor expression after spinal transection of rats: an immunohistochemical study. Neuroscience 177, 114–126. doi: 10.1016/j.neuroscience.2010.12.062, PMID: 21211552

[ref32] Kong X.-Y. Wienecke J. Hultborn H. Zhang M. (2010). Robust upregulation of serotonin 2A receptors after chronic spinal transection of rats: an immunohistochemical study. Brain Res. 1320, 60–68. doi: 10.1016/j.brainres.2010.01.030, PMID: 20085755

[ref33] Kugaya A. Epperson C. N. Zoghbi S. Van Dyck C. H. Hou Y. Fujita M.. (2003). Increase in prefrontal cortex serotonin_2A_ receptors following estrogen treatment in postmenopausal women. AJP 160, 1522–1524. doi: 10.1176/appi.ajp.160.8.1522, PMID: 12900319

[ref34] Lee R. H. Heckman C. J. (1998). Bistability in spinal motoneurons in vivo: systematic variations in rhythmic firing patterns. J. Neurophysiol. 80, 572–582. doi: 10.1152/jn.1998.80.2.572 9705451

[ref35] Lee R. H. Heckman C. J. (1999). Enhancement of bistability in spinal motoneurons in vivo by the noradrenergic α _1_ agonist methoxamine. J. Neurophysiol. 81, 2164–2174. doi: 10.1152/jn.1999.81.5.2164 10322057

[ref36] Li Q. Nakadate K. Tanaka-Nakadate S. Nakatsuka D. Cui Y. Watanabe Y. (2004). Unique expression patterns of 5-HT_2A_ and 5-HT_2C_ receptors in the rat brain during postnatal development: western blot and immunohistochemical analyses. J. Comp. Neurol. 469, 128–140. doi: 10.1002/cne.11004, PMID: 14689478

[ref37] Lin S. Li Y. Lucas-Osma A. M. Hari K. Stephens M. J. Singla R.. (2019). Locomotor-related V3 interneurons initiate and coordinate muscles spasms after spinal cord injury. J. Neurophysiol. 121, 1352–1367. doi: 10.1152/jn.00776.2018, PMID: 30625014 PMC6485742

[ref38] Lindsay A. D. Feldman J. L. (1993). Modulation of respiratory activity of neonatal rat phrenic motoneurones by serotonin. J. Physiol. 461, 213–233. doi: 10.1113/jphysiol.1993.sp019510, PMID: 8350262 PMC1175254

[ref39] Lyon M. F. (1961). Gene action in the X-chromosome of the mouse (*Mus musculus* L.). Nature 190, 372–373. doi: 10.1038/190372a0, PMID: 13764598

[ref40] Mahrous A. Birch D. Heckman C. J. Tysseling V. (2024). Muscle spasms after spinal cord injury stem from changes in motoneuron excitability and synaptic inhibition, not synaptic excitation. J. Neurosci. 44:e1695232023. doi: 10.1523/JNEUROSCI.1695-23.2023, PMID: 37949656 PMC10851678

[ref41] Mahrous A. A. Elbasiouny S. M. (2017). SK channel inhibition mediates the initiation and amplitude modulation of synchronized burst firing in the spinal cord. J. Neurophysiol. 118, 161–175. doi: 10.1152/jn.00929.2016, PMID: 28356481 PMC5494358

[ref42] Majczyński H. Cabaj A. M. Jordan L. M. Sławińska U. (2020). Contribution of 5-HT2 receptors to the control of the spinal locomotor system in intact rats. Front. Neural Circuits 14:14. doi: 10.3389/fncir.2020.00014, PMID: 32425760 PMC7212388

[ref43] Mancuso R. Oliván S. Osta R. Navarro X. (2011). Evolution of gait abnormalities in SOD1G93A transgenic mice. Brain Res. 1406, 65–73. doi: 10.1016/j.brainres.2011.06.033, PMID: 21733494

[ref44] Moalem G. Leibowitz–Amit R. Yoles E. Mor F. Cohen I. R. Schwartz M. (1999). Autoimmune T cells protect neurons from secondary degeneration after central nervous system axotomy. Nat. Med. 5, 49–55. doi: 10.1038/4734, PMID: 9883839

[ref45] Mosher T. Hayes D. Greenshaw A. (2005). Differential effects of 5-HT2C receptor ligands on place conditioning and locomotor activity in rats. Eur. J. Pharmacol. 515, 107–116. doi: 10.1016/j.ejphar.2005.03.041, PMID: 15896731

[ref46] Murray K. C. Nakae A. Stephens M. J. Rank M. D’Amico J. Harvey P. J.. (2010). Recovery of motoneuron and locomotor function after spinal cord injury depends on constitutive activity in 5-HT2C receptors. Nat. Med. 16, 694–700. doi: 10.1038/nm.2160 20512126 PMC3107820

[ref47] Murray K. C. Stephens M. J. Ballou E. W. Heckman C. J. Bennett D. J. (2011a). Motoneuron excitability and muscle spasms are regulated by 5-HT_2B_ and 5-HT_2C_ receptor activity. J. Neurophysiol. 105, 731–748. doi: 10.1152/jn.00774.2010, PMID: 20980537 PMC3059173

[ref48] Murray K. C. Stephens M. J. Rank M. D’Amico J. Gorassini M. A. Bennett D. J. (2011b). Polysynaptic excitatory postsynaptic potentials that trigger spasms after spinal cord injury in rats are inhibited by 5-HT_1B_ and 5-HT_1F_ receptors. J. Neurophysiol. 106, 925–943. doi: 10.1152/jn.01011.2010 21653728 PMC3154834

[ref49] Nebuka M. Ohmura Y. Izawa S. Bouchekioua Y. Nishitani N. Yoshida T.. (2020). Behavioral characteristics of 5-HT2C receptor knockout mice: locomotor activity, anxiety-, and fear memory-related behaviors. Behav. Brain Res. 379:112394. doi: 10.1016/j.bbr.2019.112394, PMID: 31786274

[ref50] Oydanich M. Babici D. Zhang J. Rynecki N. Vatner D. E. Vatner S. F. (2019). Mechanisms of sex differences in exercise capacity. Am. J. Phys. Regul. Integr. Comp. Phys. 316, R832–R838. doi: 10.1152/ajpregu.00394.2018, PMID: 31017810 PMC6734069

[ref51] Papaneophytou C. P. Georgiou E. Karaiskos C. Sargiannidou I. Markoullis K. Freidin M. M.. (2018). Regulatory role of oligodendrocyte gap junctions in inflammatory demyelination. Glia 66, 2589–2603. doi: 10.1002/glia.23513, PMID: 30325069 PMC6519212

[ref52] Parajulee A. Kim K. (2023). Structural studies of serotonin receptor family. BMB Rep. 56, 527–536. doi: 10.5483/BMBRep.2023-0147, PMID: 37817438 PMC10618075

[ref53] Popova N. K. Amstislavskaya T. G. (2002). 5-HT_2A_ and 5-HT_2C_ serotonin receptors differentially modulate mouse sexual arousal and the hypothalamo-pituitary-testicular response to the presence of a female. Neuroendocrinology 76, 28–34. doi: 10.1159/000063681, PMID: 12097814

[ref54] Ren L.-Q. Wienecke J. Chen M. Møller M. Hultborn H. Zhang M. (2013). The time course of serotonin 2C receptor expression after spinal transection of rats: an immunohistochemical study. Neuroscience 236, 31–46. doi: 10.1016/j.neuroscience.2012.12.063, PMID: 23337537

[ref55] Sqalli-Houssaini Y. Cazalets J.-R. (2000). Noradrenergic control of locomotor networks in the in vitro spinal cord of the neonatal rat. Brain Res. 852, 100–109. doi: 10.1016/S0006-8993(99)02219-2, PMID: 10661501

[ref56] Stamm S. Gruber S. B. Rabchevsky A. G. Emeson R. B. (2017). The activity of the serotonin receptor 2C is regulated by alternative splicing. Hum. Genet. 136, 1079–1091. doi: 10.1007/s00439-017-1826-3, PMID: 28664341 PMC5873585

[ref57] Sumner B. E. H. Fink G. (1995). Estrogen increases the density of 5-Hydroxytryptamine2A receptors in cerebral cortex and nucleus accumbens in the female rat. J. Steroid Biochem. Mol. Biol. 54, 15–20. doi: 10.1016/0960-0760(95)00075-B, PMID: 7632610

[ref58] Tecott L. H. Sun L. M. Akana S. F. Strack A. M. Lowenstein D. H. Dallman M. F.. (1995). Eating disorder and epilepsy in mice lacking 5-HT2C serotonin receptors. Nature 374, 542–546. doi: 10.1038/374542a0, PMID: 7700379

[ref59] Tenan M. S. Peng Y.-L. Hackney A. C. Griffin L. (2013). Menstrual cycle mediates vastus Medialis and vastus Medialis oblique muscle activity. Med. Sci. Sports Exerc. 45, 2151–2157. doi: 10.1249/MSS.0b013e318299a69d, PMID: 23657168

[ref60] Thorstensen J. R. Taylor J. L. Kavanagh J. J. (2021). Human corticospinal-motoneuronal output is reduced with 5-HT_2_ receptor antagonism. J. Neurophysiol. 125, 1279–1288. doi: 10.1152/jn.00698.2020, PMID: 33596722

[ref61] Tysseling V. M. Klein D. A. Imhoff-Manuel R. Manuel M. Heckman C. J. Tresch M. C. (2017). Constitutive activity of 5-HT_2C_ receptors is present after incomplete spinal cord injury but is not modified after chronic SSRI or baclofen treatment. J. Neurophysiol. 118, 2944–2952. doi: 10.1152/jn.00190.2017, PMID: 28877964 PMC5686237

[ref62] Zhang J. Engel J. A. Jackson D. M. Johansson C. Svensson L. (1997). (−)Alprenolol potentiates the disrupting effects of dizocilpine on sensorimotor function in the rat. Psychopharmacology 132, 281–288. doi: 10.1007/s002130050346, PMID: 9292628

[ref63] Zhang H. Li L. Zhang X. Ru G. Zang W. (2024). Role of the dorsal raphe nucleus in pain processing. Brain Sci. 14:982. doi: 10.3390/brainsci14100982, PMID: 39451996 PMC11506261

