# Supplementary figures and images for: Loss of 5-HT2C receptor function alters motor behavior in male and female mice with and without spinal cord injury
Source: Front Neural Circuits. 2025 Sep 29;19:1681120. doi: 10.3389/fncir.2025.1681120 (PMC12515959; doi:10.3389/fncir.2025.1681120)

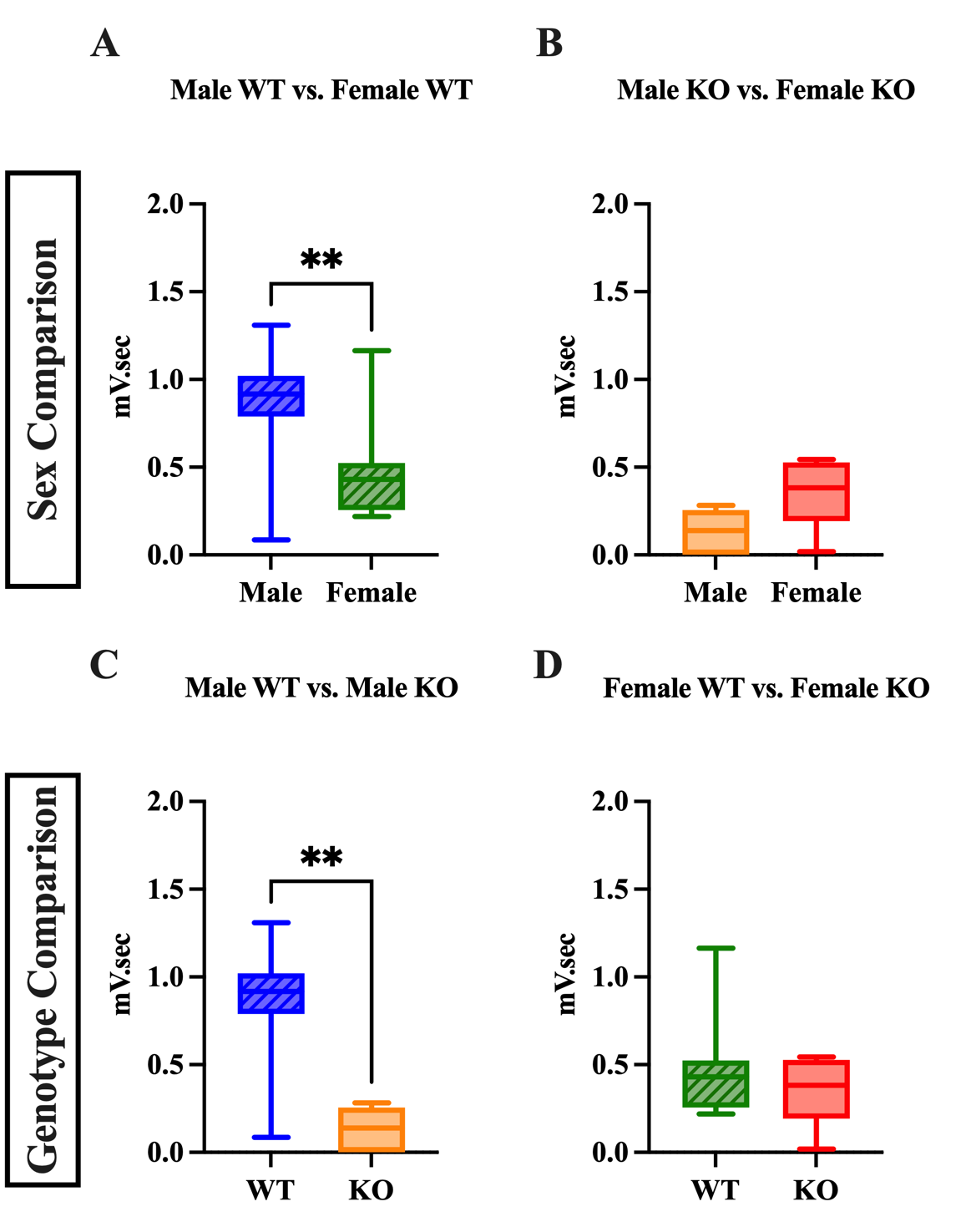

Supplement: SUPPLEMENTARY FIGURE 1 — Male KO and female WT mice exhibit reduced spasm-like activity in LLR 5 (s) compared to male WT mice. Box plots compared LLR (5 s) activity between male and female WT mice (A), male and female KO mice (B), male WT and male KO mice (C), and female WT and female KO mice (D). An unpaired t-test was used for comparison between male and female mice; all other comparisons used a Mann-Whitney test due to non-normal data distributions. Male WT (n = 11), female WT (n = 16), male KO (n = 6), and female KO (n = 7) mice were included in all analyses. ****p < 0.0001, ***p < 0.001, **p < 0.01, *p < 0.05. [file Image_1.tiff]

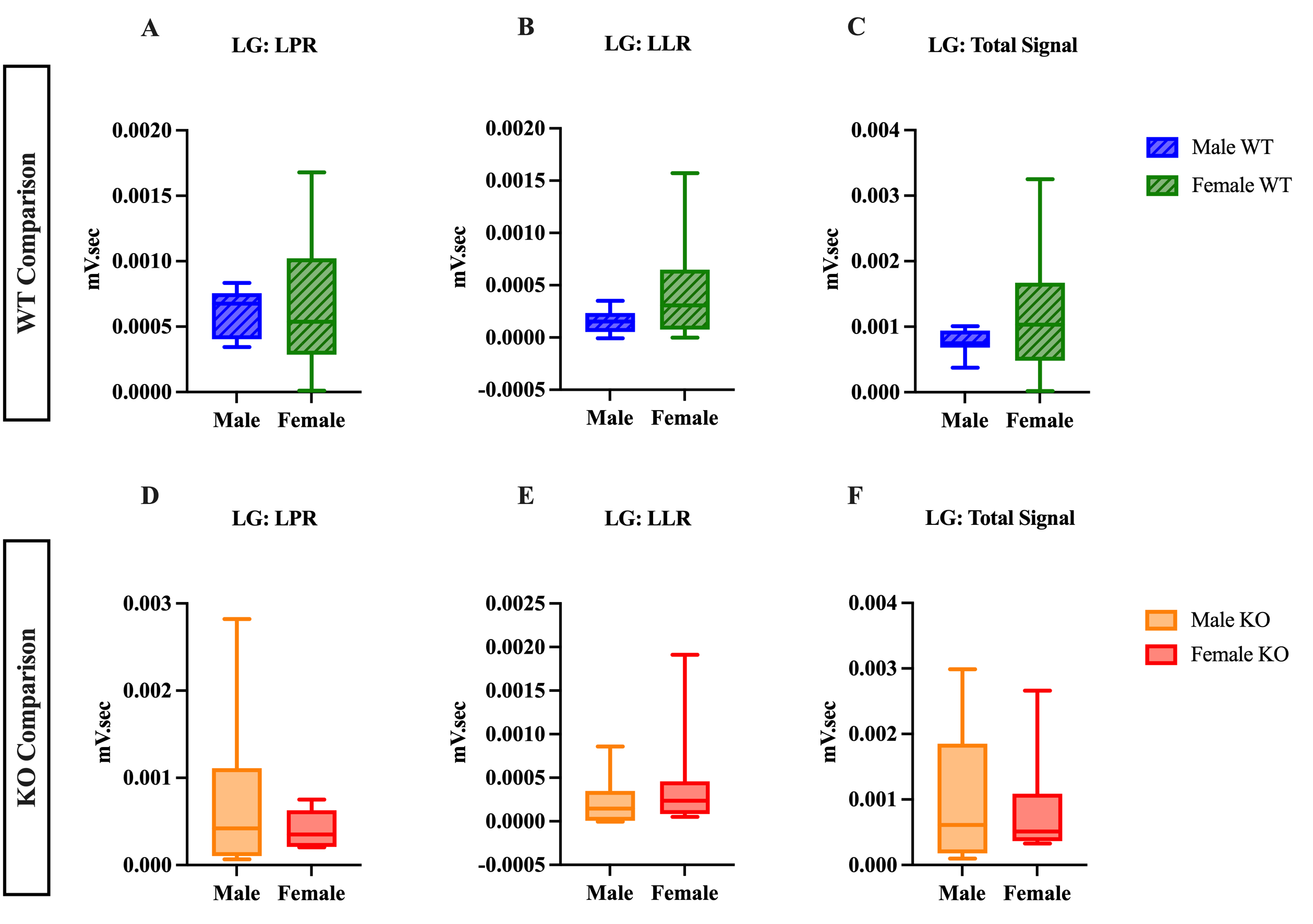

Supplement: SUPPLEMENTARY FIGURE 2 — No significant differences in hyperreflexia in the LG muscle between sexes within each genotype. Box plots illustrating sex-specific differences in LPR (A,D), LLR (B,E), and total signal (C,F) of the LG muscle between male and female WT mice (upper panel), and male and female KO mice (lower panel). Male WT (n = 8), male KO (n = 8), female WT (n = 11), and female KO (n = 9) were included in the analyses. An unpaired t-test was used for comparison of LPR and total signal between male and female WT mice and LLR between male and female KO mice; a Mann-Whitney test was used for all other comparisons due to non-normal data distributions. LLR, long-latency response; LPR, longer polysynaptic response. ****p < 0.0001, ***p < 0.001, **p < 0.01, *p < 0.05. [file Image_2.tiff]

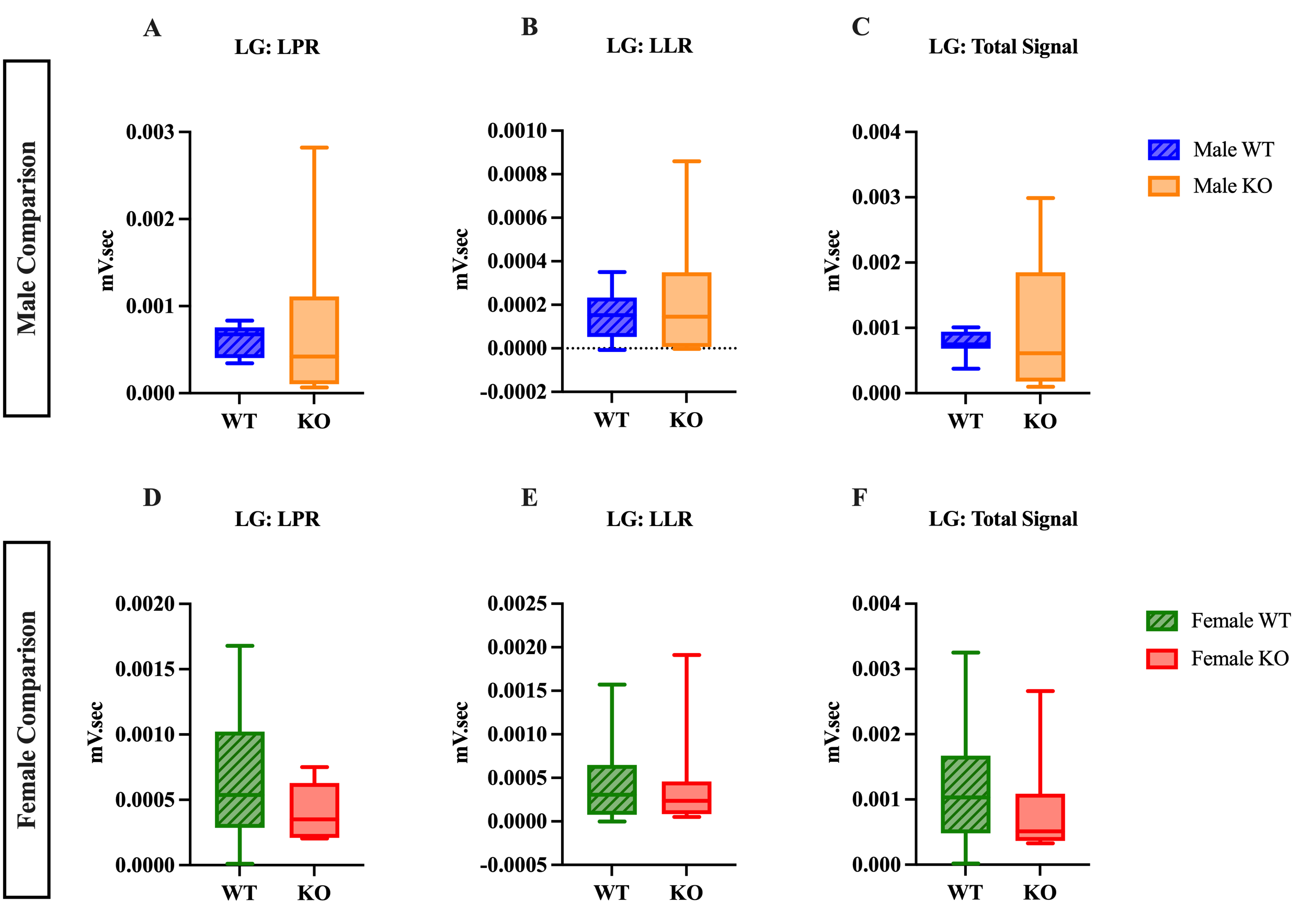

Supplement: SUPPLEMENTARY FIGURE 3 — No significant differences in hyperreflexia in the LG muscle between genotypes within each sex. Box plots illustrating genotype-specific differences in LPR (A,D), LLR (B,E), and total signal (C,F) of the LG muscle between male WT and KO mice (upper panel), and female WT and KO mice (lower panel). Male WT (n = 8), male KO (n = 8), female WT (n = 11), and female KO (n = 9) were included in the analyses. An unpaired t-test was used for comparison of LPR between female WT and KO mice and total signal between male WT and KO mice; a Mann-Whitney test was used for all other comparisons due to non-normal data distributions. LLR, long-latency response; LPR, longer polysynaptic response. ****p < 0.0001, ***p < 0.001, **p < 0.01, *p < 0.05. [file Image_3.tiff]

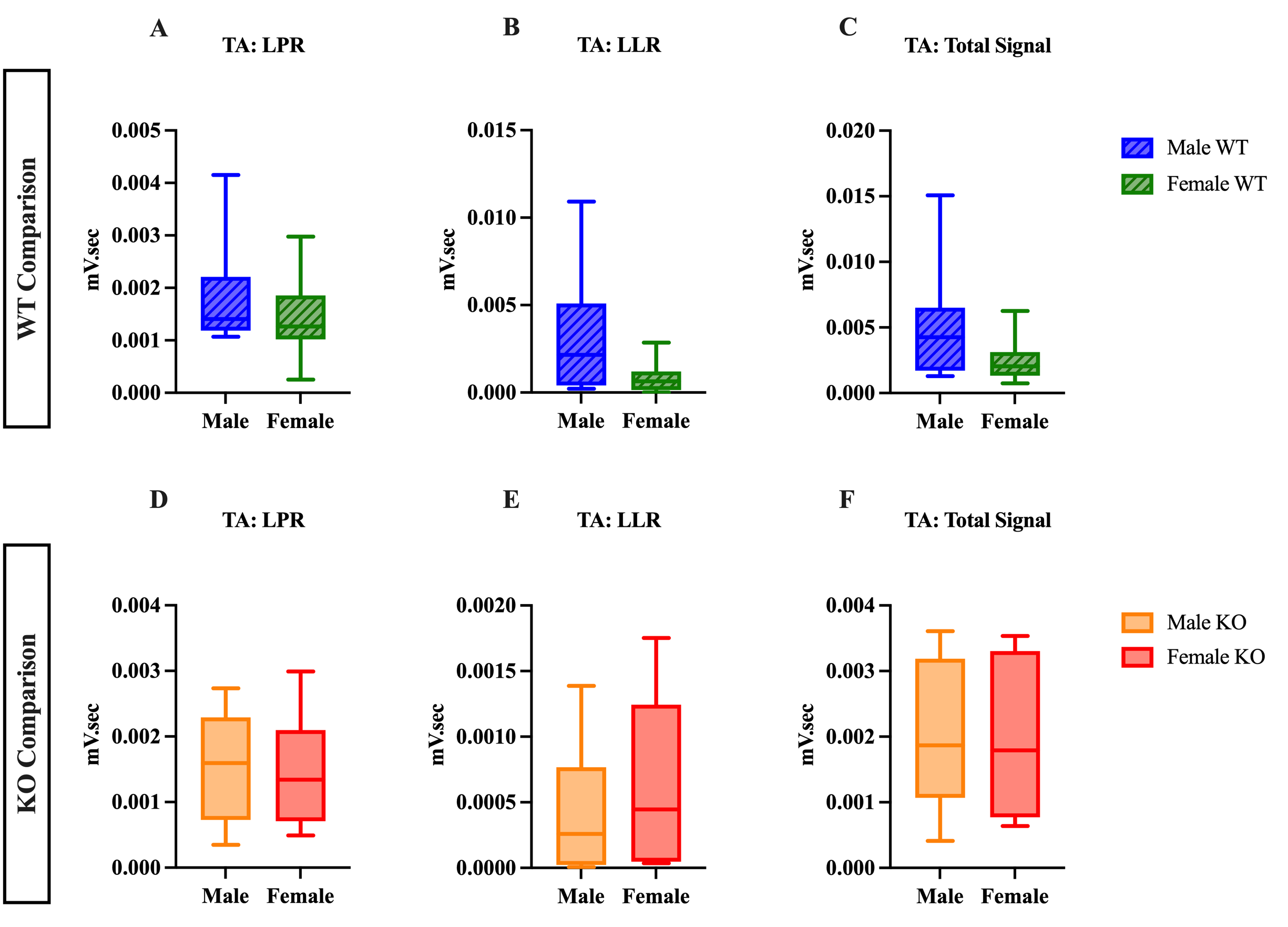

Supplement: SUPPLEMENTARY FIGURE 4 — No difference in hyperreflexia in the TA muscle between sexes within each genotype. Box plots illustrating sex-specific differences in LPR (A,D), LLR (B,E), and total signal (C,F) of the TA muscle between male and female WT mice (upper panel), and male and female KO mice (lower panel). Male WT (n = 8), male KO (n = 8), female WT (n = 11), and female KO (n = 9) were included in the analyses. An unpaired t-test was used for comparison of LPR, LLR, and total signal between male and female KO mice; a Mann-Whitney test was used for all other comparisons due to non-normal data distributions. LLR, long-latency response; LPR, longer polysynaptic response. ****p < 0.0001, ***p < 0.001, **p < 0.01, *p < 0.05. [file Image_4.tiff]

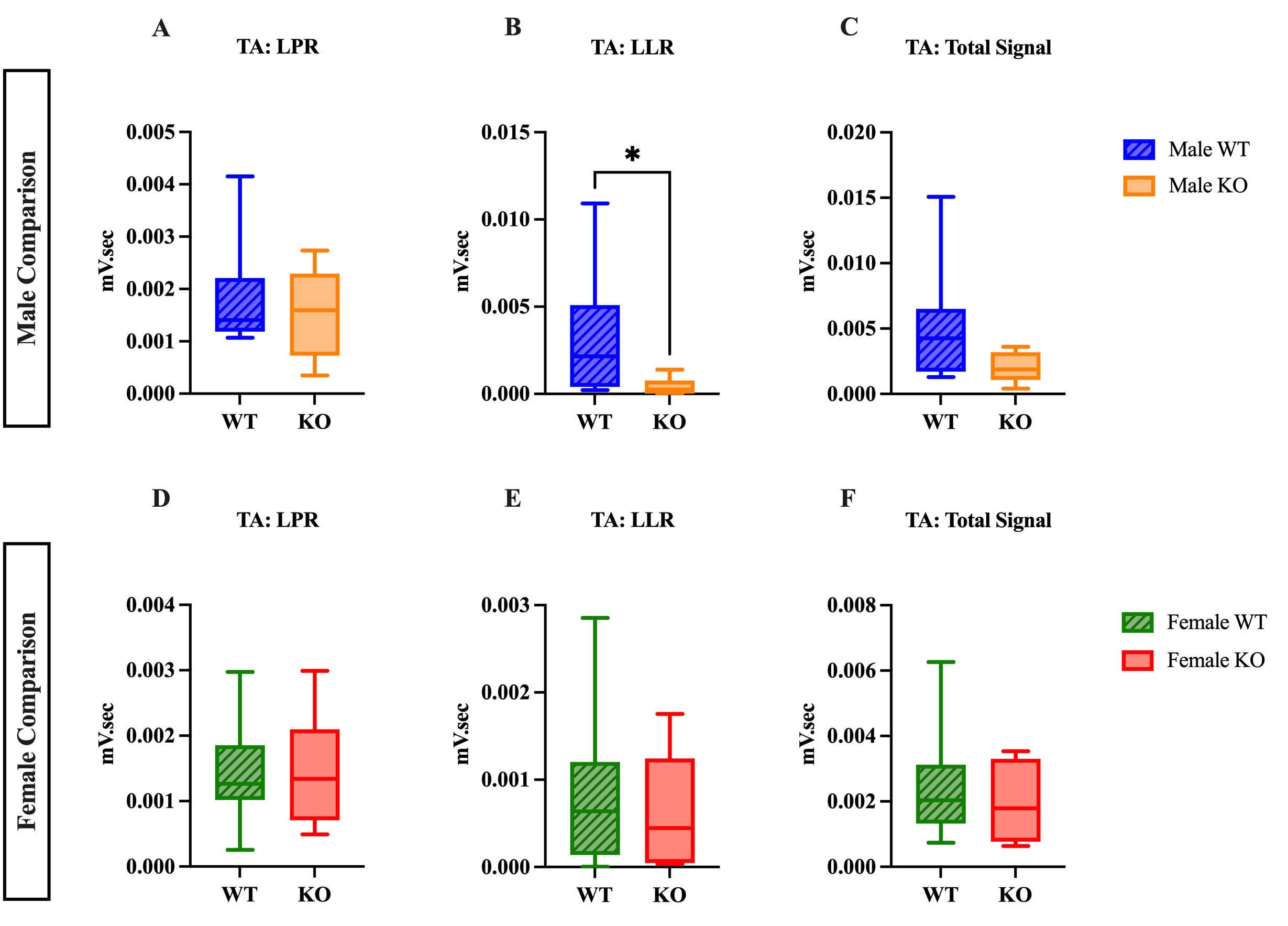

Supplement: SUPPLEMENTARY FIGURE 5 — Only male KO mice exhibit reduced hyperreflexia compared to male WT mice in the LLR of the TA muscle. Box plots illustrating genotype-specific differences in LPR (A,D), LLR (B,E), and total signal (C,F) of the TA muscle between male WT and KO mice (upper panel), and female WT and KO mice (lower panel). Male WT (n = 8), male KO (n = 8), female WT (n = 11), and female KO (n = 9) were included in the analyses. An unpaired t-test was used for comparison of LPR, LLR, and total signal between female WT and KO mice; a Mann-Whitney test was used for all other comparisons due to non-normal data distributions. LLR, long-latency response; LPR, longer polysynaptic response. ****p < 0.0001, ***p < 0.001, **p < 0.01, *p < 0.05. [file Image_5.tiff]

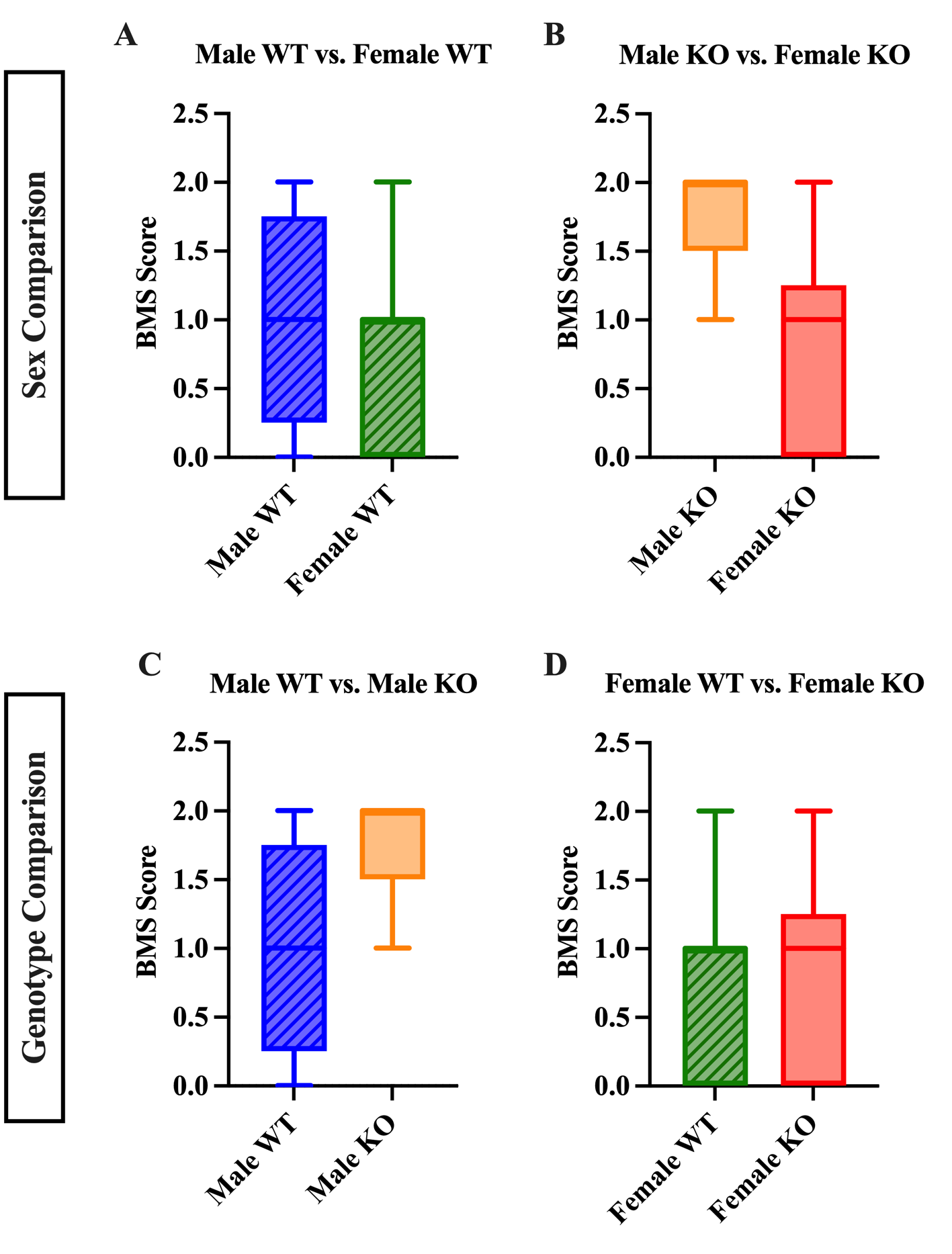

Supplement: SUPPLEMENTARY FIGURE 6 — No significant difference in averaged Basso Mouse Scale (BMS) scores at chronicity (weeks 8–10). Box plots illustrating sex-specific differences in BMS between male and female WT mice (A) and male and female KO mice (B), as well as genotype-specific differences between male WT and KO mice (C) and female WT and KO mice (D). Male WT (n = 8), male KO (n = 5), female WT (n = 7), and female KO (n = 6) were included in the analyses. An unpaired t-test was used for comparison of male and female WT mice and female WT and KO mice; a Mann-Whitney test was used for all other comparisons due to non-normal data distributions. ****p < 0.0001, ***p < 0.001, **p < 0.01, *p < 0.05. [file Image_6.tiff]

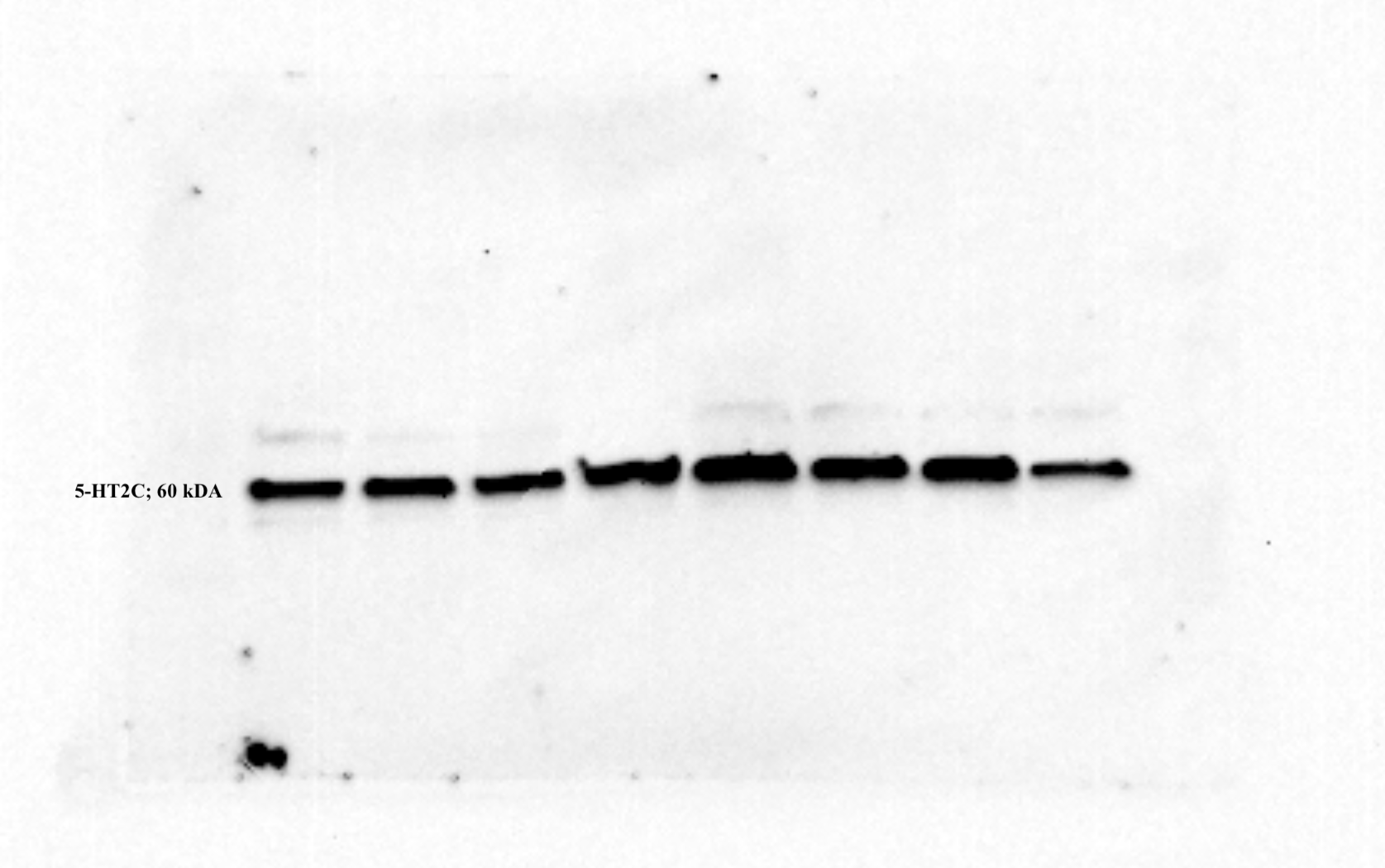

Supplement: SUPPLEMENTARY FIGURE 7 — Full western blot results of 5-HT2C as shown in Figure 6P. Western blot analysis of 5-HT2C expression in uninjured female KO mice (bands 1 – 4) and uninjured female WT mice (bands 5 – 8). [file Image_7.tif]

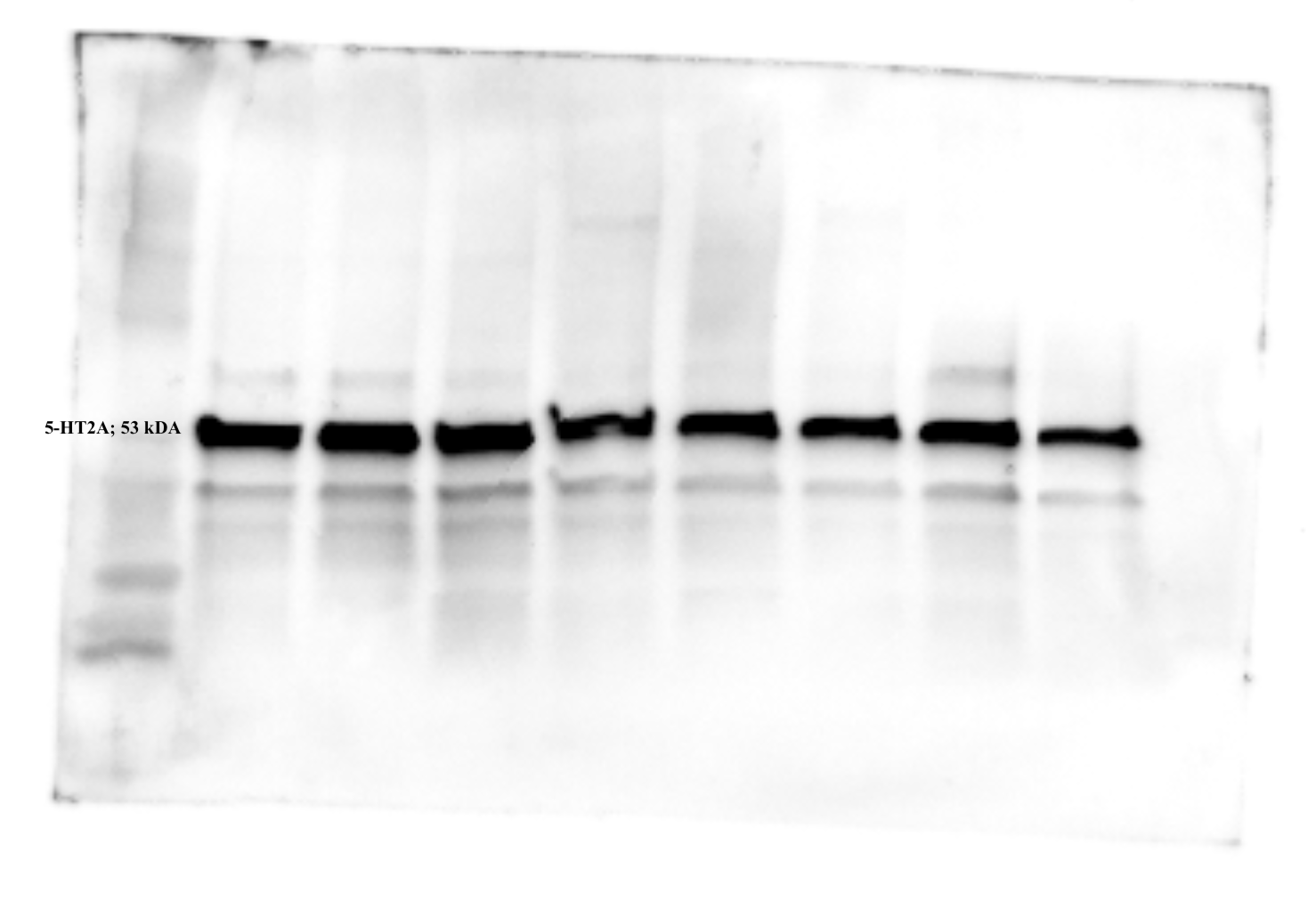

Supplement: SUPPLEMENTARY FIGURE 8 — Full western blot results of 5-HT2A as shown in Figure 6P. Western blot analysis of 5-HT2A expression in uninjured female KO mice (bands 1 – 4) and uninjured female WT mice (bands 5 – 8). [file Image_8.tif]

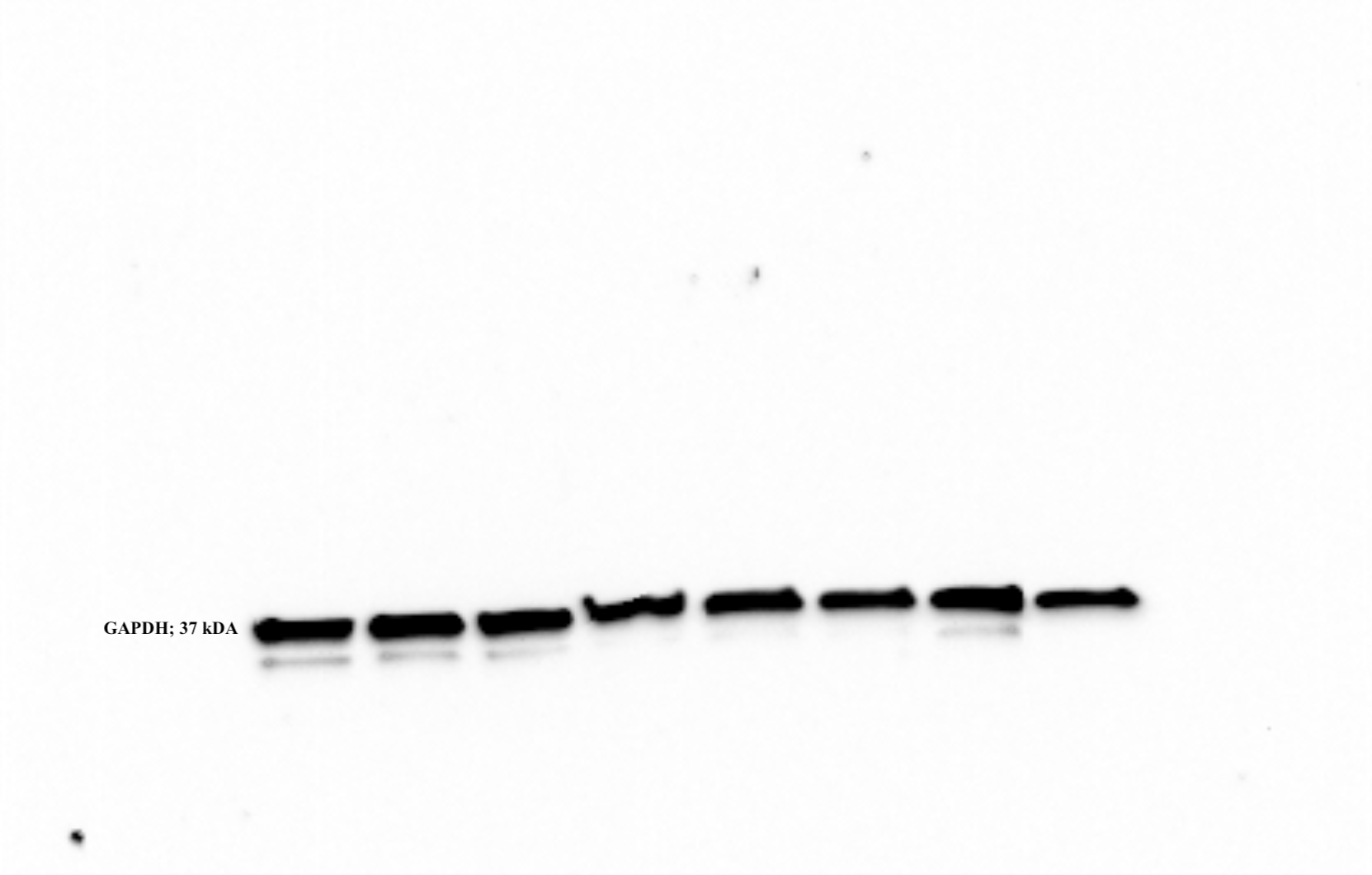

Supplement: SUPPLEMENTARY FIGURE 9 — Full western blot results of GAPDH as shown in Figure 6P. Western blot analysis of GAPDH expression in uninjured female KO mice (bands 1 – 4) and uninjured female WT mice (bands 5 – 8). [file Image_9.tif]

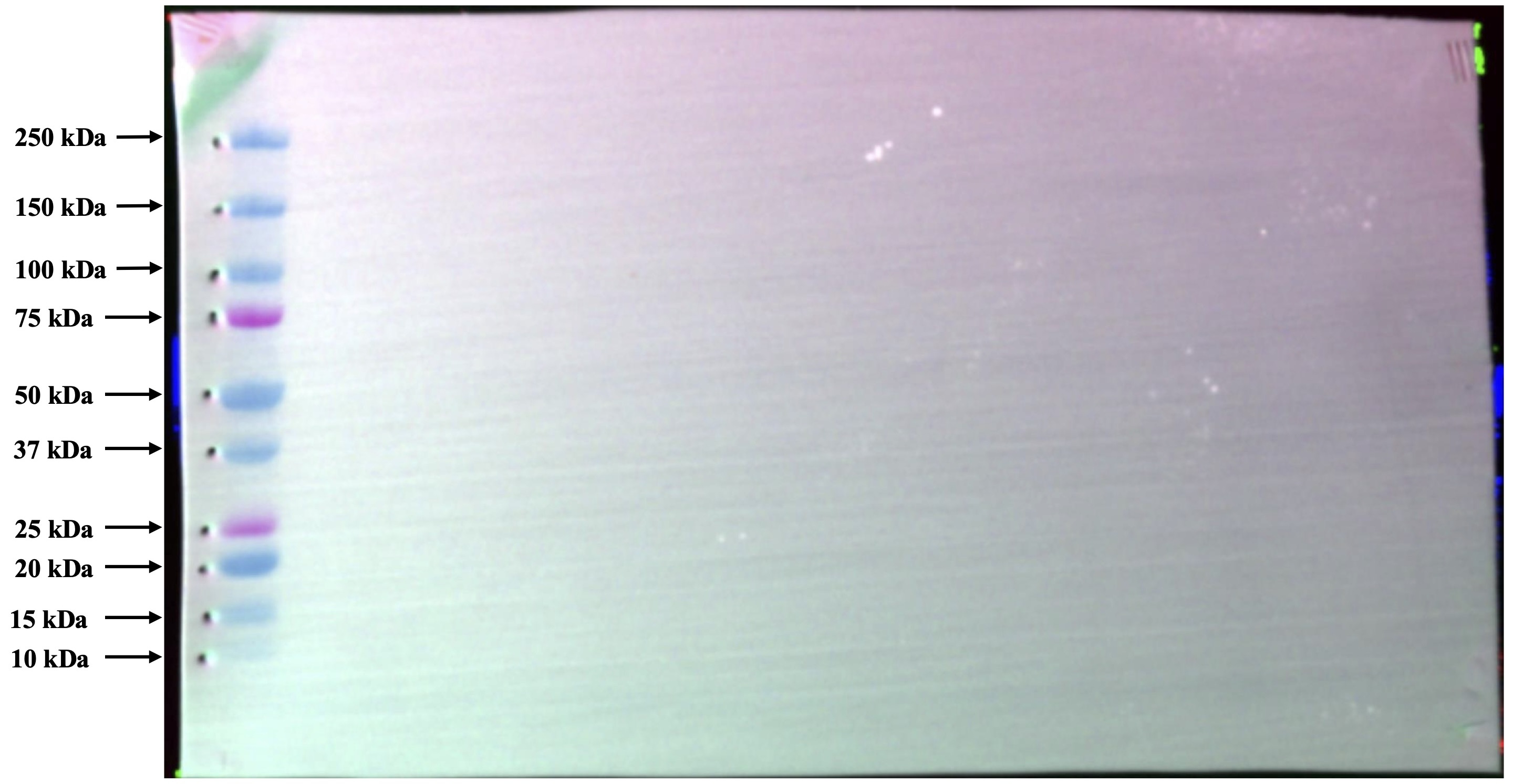

Supplement: SUPPLEMENTARY FIGURE 10 — Full western blot results of Precision Plus Protein Dual Color Standard. The protein ladder was used in all western blots in lane 1 to visually assess and confirm the molecular weight of chemiluminescent bands from the protein of interest. Molecular weights (kDa) are listed on the left-hand side of each marker. [file Image_10.jpeg]
