# Supplementary material for: Loss of 5-HT2C receptor function alters motor behavior in male and female mice with and without spinal cord injury
Source: Front Neural Circuits. 2025 Sep 29;19:1681120. doi: 10.3389/fncir.2025.1681120 (PMC12515959; doi:10.3389/fncir.2025.1681120)
Supplement: Supplementary file 11 [file Table_1.docx]

Supplementary Material

# Supplementary Table 1. Complete list of male WT mice (mouse numbers 1 – 26) used in each experiment, and the total n of the group (left) and the total n of each experiment (right) is shown on the bottom row.
