# Supplementary material for: Loss of 5-HT2C receptor function alters motor behavior in male and female mice with and without spinal cord injury
Source: Front Neural Circuits. 2025 Sep 29;19:1681120. doi: 10.3389/fncir.2025.1681120 (PMC12515959; doi:10.3389/fncir.2025.1681120)
Supplement: Supplementary file 15 [file Table_5.docx]

Supplementary Material

# Supplementary Table 5. Complete list of parametric western blot statistical results.

| **Parameter** | | **M** | **SD** | **t** | **df** | **95% CI** | ***P*-Value** |
| --- | --- | --- | --- | --- | --- | --- | --- |
| **Effect of Injury** | Uninjured Male WT (L, 2C) vs. | 1.362 | 0.5117 | 0.3507 | 6 | -1.008 to 0.7553 | .7378 |
|  | Injured Male WT (L, 2C) | 1.236 | 0.5074 |  |  |  |  |
|  | Uninjured Male WT (S, 2C) vs. | 2.067 | 0.9579 | 2.36 | 6 | -2.303 to 0.04147 | .0563 |
|  | Injured Male WT (S, 2C) | 0.9367 | 0.01565 |  |  |  |  |
|  | Uninjured Male WT (L, 2A) vs. | 2.136 | 0.7174 | 2.467 | 6 | -2.223 to -0.009176 | .0486 |
|  | Injured Male WT (L, 2A) | 1.02 | 0.5512 |  |  |  |  |
|  | Uninjured Male WT (S, 2A) vs. | 1.871 | 1.234 | 1.984 | 6 | -2.759 to 0.2883 | .0945 |
|  | Injured Male WT (S, 2A) | 0.6357 | 0.1701 |  |  |  |  |
|  | Uninjured Female WT (L, 2C) vs. | 1.132 | 0.2898 | 2.879 | 6 | -0.8055 to -0.06533 | .0281 |
|  | Injured Female WT (L, 2C) | 0.6969 | 0.08657 |  |  |  |  |
|  | Uninjured Female WT (S, 2C) vs. | 1.546 | 0.371 | 3.33 | 6 | -1.439 to -0.2201 | .0158 |
|  | Injured Female WT (S, 2C) | 0.7164 | 0.3325 |  |  |  |  |
|  | Uninjured Female WT (L, 2A) vs. | 0.2051 | 0.02674 | 11.81 | 6 | 0.5326 to 0.8110 | < .0001 |
|  | Injured Female WT (L, 2A) | 0.8769 | 0.1106 |  |  |  |  |
|  | Uninjured Female WT (S, 2A) vs. | 0.9368 | 0.1122 | 1.407 | 6 | -0.5050 to 0.1362 | .209 |
|  | Injured Female WT (S, 2A) | 0.7524 | 0.2368 |  |  |  |  |
|  | Uninjured Male KO (L, 2A) vs. | 1.125 | 0.1279 | 0.5102 | 6 | -0.4540 to 0.2973 | .6281 |
|  | Injured Male KO (L, 2A) | 1.047 | 0.2791 |  |  |  |  |
|  | Uninjured Male KO (S, 2A) vs. | 1.126 | 0.4632 | 1.255 | 6 | -1.029 to 0.3315 | .2563 |
|  | Injured Male KO (S, 2A) | 0.7774 | 0.3075 |  |  |  |  |
|  | Uninjured Female KO (L, 2A) vs. | 0.298 | 0.0671 | 14.14 | 6 | 0.6119 to 0.8681 | < .0001 |
|  | Injured Female KO (L, 2A) | 1.038 | 0.08038 |  |  |  |  |
|  | Uninjured Female KO (S, 2A) vs. | 1.509 | 0.7204 | 1.565 | 6 | -1.509 to 0.3318 | .1687 |
|  | Injured Female KO (S, 2A) | 0.9204 | 0.216 |  |  |  |  |
| **Effect of Genotype** | Uninjured Male WT (L, 2A) vs. | 2.136 | 0.7174 | 2.775 | 6 | -1.903 to -0.1196 | .0322 |
|  | Uninjured Male KO (L, 2A) | 1.125 | 0.1279 |  |  |  |  |
|  | Uninjured Male WT (S, 2A) vs. | 1.871 | 1.234 | 1.13 | 6 | -2.357 to 0.8674 | .3015 |
|  | Uninjured Male KO (S, 2A) | 1.126 | 0.4632 |  |  |  |  |
|  | Injured Male WT (L, 2A) vs. | 1.02 | 0.5512 | 0.08591 | 6 | -0.7294 to 0.7825 | .9343 |
|  | Injured Male KO (L, 2A) | 1.047 | 0.2791 |  |  |  |  |
|  | Injured Male WT (S, 2A) vs. | 0.6357 | 0.1701 | 0.8066 | 6 | -0.2882 to 0.5716 | .4507 |
|  | Injured Male KO (S, 2A) | 0.7774 | 0.3075 |  |  |  |  |
|  | Uninjured Female WT (L, 2A) vs. | 0.2051 | 0.02674 | 2.572 | 6 | 0.004535 to 0.1813 | .0422 |
|  | Uninjured Female KO (L, 2A) | 0.298 | 0.0671 |  |  |  |  |
|  | Uninjured Female WT (S, 2A) vs. | 0.9368 | 0.1122 | 1.569 | 6 | -0.3200 to 1.464 | .1677 |
|  | Uninjured Female KO (S, 2A) | 1.509 | 0.7204 |  |  |  |  |
|  | Injured Female WT (L, 2A) vs. | 0.8769 | 0.1106 | 2.358 | 6 | -0.006086 to 0.3284 | .0564 |
|  | Injured Female KO (L, 2A) | 1.038 | 0.08038 |  |  |  |  |
|  | Injured Female WT (S, 2A) vs. | 0.7524 | 0.2368 | 1.048 | 6 | -0.2241 to 0.5601 | .3348 |
|  | Injured Female KO (S, 2A) | 0.9204 | 0.216 |  |  |  |  |
| **Effect of Sex** | Uninjured Male WT (L, 2C) vs. | 1.362 | 0.5117 | 0.7806 | 6 | -0.9490 to 0.4899 | .4647 |
|  | Uninjured Female WT (L, 2C) | 1.132 | 0.2898 |  |  |  |  |
|  | Uninjured Male WT (S, 2C) vs. | 2.067 | 0.9579 | 1.015 | 6 | -1.778 to 0.7355 | .3493 |
|  | Uninjured Female WT (S, 2C) | 1.546 | 0.371 |  |  |  |  |
|  | Injured Male WT (S, 2C) vs. | 1.236 | 0.5074 | 2.093 | 6 | -1.168 to 0.09119 | .0813 |
|  | Injured Female WT (S, 2C) | 0.6969 | 0.08657 |  |  |  |  |
|  | Injured Male WT (S, 2C) vs. | 0.9367 | 0.01565 | 1.323 | 6 | -0.6275 to 0.1870 | .2339 |
|  | Injured Female WT (S, 2C) | 0.7164 | 0.3325 |  |  |  |  |
|  | Uninjured Male WT (L, 2A) vs. | 2.136 | 0.7174 | 5.38 | 6 | -2.810 to -1.053 | .0017 |
|  | Uninjured Female WT (L, 2A) | 0.2051 | 0.02674 |  |  |  |  |
|  | Uninjured Male WT (S, 2A) vs. | 1.871 | 1.234 | 1.508 | 6 | -2.450 to 0.5814 | .1822 |
|  | Uninjured Female WT (S, 2A) | 0.9368 | 0.1122 |  |  |  |  |
|  | Injured Male WT (L, 2A) vs. | 1.02 | 0.5512 | 0.5102 | 6 | -0.8312 to 0.5444 | .6281 |
|  | Injured Female WT (L, 2A) | 0.8769 | 0.1106 |  |  |  |  |
|  | Injured Male WT (L, 2A) vs. | 0.6357 | 0.1701 | 0.801 | 6 | -0.2399 to 0.4734 | .4537 |
|  | Injured Female WT (L, 2A) | 0.7524 | 0.2368 |  |  |  |  |
|  | Uninjured Male KO (L, 2A) vs. | 1.125 | 0.1279 | 11.46 | 6 | -1.004 to -0.6505 | < .0001 |
|  | Uninjured Female KO (L, 2A) | 0.298 | 0.0671 |  |  |  |  |
|  | Uninjured Male KO (S, 2A) vs. | 1.126 | 0.4632 | 0.8936 | 6 | -0.6652 to 1.431 | .406 |
|  | Uninjured Female KO (S, 2A) | 1.509 | 0.7204 |  |  |  |  |
|  | Injured Male KO (L, 2A) vs. | 1.047 | 0.2791 | 0.06054 | 6 | -0.3642 to 0.3466 | .9537 |
|  | Injured Female KO (L, 2A) | 1.038 | 0.8038 |  |  |  |  |
|  | Injured Male KO (S, 2A) vs. | 0.7774 | 0.3075 | 0.7614 | 6 | -0.3167 to 0.6028 | .4753 |
|  | Injured Female KO (S, 2A) | 0.9204 | 0.216 |  |  |  |  |
| **Effect of Receptor Distribution** | Uninjured Male WT (L, 2C) vs. | 2.136 | 0.7174 | 1.758 | 6 | -1.853 to 0.3036 | .1293 |
|  | Uninjured Male WT (L, 2A) | 1.362 | 0.5117 |  |  |  |  |
|  | Uninjured Male WT (L, 2C) vs. | 1.871 | 1.234 | 0.2513 | 6 | -1.715 to 2.107 | .8099 |
|  | Uninjured Male WT (Sacral, 2A) | 2.067 | 0.9579 |  |  |  |  |
|  | Injured Male WT (L, 2C) vs. | 1.02 | 0.5512 | 0.5746 | 6 | -0.7014 to 1.132 | .5865 |
|  | Injured Male WT (L, 2A) | 1.236 | 0.5074 |  |  |  |  |
|  | Uninjured Female WT (L, 2C) vs. | 0.2051 | 0.02674 | 6.372 | 6 | 0.5712 to 1.283 | .0007 |
|  | Uninjured Female WT (L, 2A) | 1.132 | 0.2898 |  |  |  |  |
|  | Uninjured Female WT (L, 2C) vs. | 0.9368 | 0.1122 | 3.143 | 6 | 0.1350 to 1.083 | .02 |
|  | Uninjured Female WT (L, 2A) | 1.546 | 0.371 |  |  |  |  |
|  | Injured Female WT (L, 2C) vs. | 0.8769 | 0.1106 | 2.562 | 6 | -0.3517 to -0.008116 | .0428 |
|  | Injured Female WT (L, 2A) | 0.6969 | 0.08657 |  |  |  |  |
|  | Injured Female WT (S, 2C) vs. | 0.7524 | 0.2368 | 0.1764 | 6 | -0.5354 to 0.4634 | .8658 |
|  | Injured Female WT (S, 2A) | 0.7164 | 0.3325 |  |  |  |  |
| **Effect of Spinal Section Distribution** | Uninjured Male WT (L, 2C) vs. | 1.362 | 0.5117 | 1.299 | 6 | -0.6232 to 2.034 | .2416 |
|  | Uninjured Male WT (S, 2C) | 2.067 | 0.9579 |  |  |  |  |
|  | Uninjured Male WT (L, 2A) vs. | 2.136 | 0.7174 | 0.3719 | 6 | -2.011 to 1.481 | .7228 |
|  | Uninjured Male WT (S, 2A) | 1.871 | 1.234 |  |  |  |  |
|  | Injured Male WT (L, 2C) vs. | 1.236 | 0.5074 | 1.177 | 6 | -0.9199 to 0.3223 | .2837 |
|  | Injured Male WT (S, 2C) | 0.9367 | 0.01565 |  |  |  |  |
|  | Injured Male WT (L, 2A) vs. | 1.02 | 0.5512 | 1.334 | 6 | -1.090 to 0.3211 | .2307 |
|  | Injured Male WT (S, 2A) | 0.6357 | 0.1701 |  |  |  |  |
|  | Uninjured Female WT (L, 2C) vs. | 1.132 | 0.2898 | 1.757 | 6 | -0.1624 to 0.9896 | .1294 |
|  | Uninjured Female WT (S, 2C) | 1.546 | 0.371 |  |  |  |  |
|  | Uninjured Female WT (L, 2A) vs. | 0.6969 | 0.08657 | 0.1134 | 6 | -0.4009 to 0.4398 | .9134 |
|  | Uninjured Female WT (S, 2A) | 0.7164 | 0.3325 |  |  |  |  |
|  | Injured Female WT (L, 2C) vs. | 0.2051 | 0.02674 | 12.68 | 6 | 0.5906 to 0.8729 | < .0001 |
|  | Injured Female WT (S, 2C) | 0.9368 | 0.1122 |  |  |  |  |
|  | Injured Female WT (L, 2A) vs. | 0.8769 | 0.1106 | 0.9525 | 6 | -0.4442 to 0.1953 | .3776 |
|  | Injured Female WT (S, 2A) | 0.7524 | 0.2368 |  |  |  |  |
|  | Uninjured Male KO (L, 2A) vs. | 1.047 | 0.2791 | 1.298 | 6 | -0.7775 to 0.2386 | .2421 |
|  | Uninjured Male KO (S, 2A) | 0.7774 | 0.3075 |  |  |  |  |
|  | Injured Male KO (L, 2A) vs. | 1.125 | 0.1279 | 0.004141 | 6 | -0.5869 to 0.5889 | .9968 |
|  | Injured Male KO (S, 2A) | 1.126 | 0.4632 |  |  |  |  |
|  | Uninjured Female KO (L, 2A) vs. | 0.298 | 0.0671 | 3.347 | 6 | 0.3256 to 2.096 | .0155 |
|  | Uninjured Female KO (S, 2A) | 1.509 | 0.7204 |  |  |  |  |
|  | Injured Female KO (L, 2A) vs. | 1.038 | 0.08038 | 1.021 | 6 | -0.3996 to 0.1644 | .3469 |
|  | Injured Female KO (S, 2A) | 0.9204 | 0.216 |  |  |  |  |

* Sample size (n) = 4 for all mouse groups, a *t*-test was used for all comparisons. ****p < .0001, ***p < .001, **p < .01, *p < .05.
