# Supplementary material for: Loss of 5-HT2C receptor function alters motor behavior in male and female mice with and without spinal cord injury
Source: Front Neural Circuits. 2025 Sep 29;19:1681120. doi: 10.3389/fncir.2025.1681120 (PMC12515959; doi:10.3389/fncir.2025.1681120)
Supplement: Supplementary file 16 [file Table_6.docx]

Supplementary Material

# Supplementary Table 6. Complete list of non-parametric western blot statistical results.

| **Parameter** | | **M** | **SD** | **Lower 95% CI** | **Upper 95% CI** | ***P*-Value** |
| --- | --- | --- | --- | --- | --- | --- |
| **Effect of Receptor Distribution** | Injured Male WT (Sacral, 2C) vs. | 0.9367 | 0.01565 | 0.9118 | 0.9616 | .0286 |
|  | Injured Male WT (Sacral, 2A) | 0.6357 | 0.1701 | 0.365 | 0.9063 |  |

* Sample size (n) = 4 for all mouse groups; a Mann-Whitney U-test was used for all comparisons. ****p < .0001, ***p < .001, **p < .01, *p < .05.
