# Supplementary material for: Loss of 5-HT2C receptor function alters motor behavior in male and female mice with and without spinal cord injury
Source: Front Neural Circuits. 2025 Sep 29;19:1681120. doi: 10.3389/fncir.2025.1681120 (PMC12515959; doi:10.3389/fncir.2025.1681120)
Supplement: Supplementary file 17 [file Table_7.docx]

Supplementary Material

# Supplementary Table 7. Table describing which mice were used in each western blot. Lanes 2 – 5 are all KO mice, and lanes 6 – 9 are all WT mice. The sex and injury status of the mice for each specific row are shown in the last two columns on the right-hand side of the table.

#

* Sample size (n) = 4 for all mouse groups.
